# First-in-Human Phase I/II Study of INCAGN01876, a Glucocorticoid-Induced Tumor Necrosis Factor Receptor Agonist, in Patients with Advanced or Metastatic Solid Tumors

**DOI:** 10.1158/1078-0432.CCR-24-4141

**Published:** 2025-07-25

**Authors:** Omid Hamid, Dana B. Cardin, F. Stephen Hodi, Patricia LoRusso, Taha Merghoub, Roberta Zappasodi, Rachana Maniyar, John E. Janik, Maikel V.W. van der Velden, Feng Zhou, Zhiwan Dong, Xuejun Chen, James J. Harding

**Affiliations:** 1 The Angeles Clinic and Research Institute, A Cedars Sinai Affiliate, Los Angeles, California.; 2 Division of Hematology and Oncology, Department of Internal Medicine, Vanderbilt University Medical Center, Nashville, Tennessee.; 3 Dana-Farber Cancer Institute, Boston, Massachusetts.; 4 Yale Cancer Center, New Haven, Connecticut.; 5 Meyer Cancer Center, Weill Cornell Medicine, Weill Cornell Medical College, New York, New York.; 6 Incyte Corporation, Wilmington, Delaware.; 7 Incyte Biosciences International Sàrl, Morges, Switzerland.; 8 Department of Medicine, Memorial Sloan Kettering Cancer Center, New York, New York.; 9 Weill Medical College at Cornell University, New York, New York.

## Abstract

**Purpose:**

Glucocorticoid-induced tumor necrosis factor receptor–related protein (GITR) agonism in T cells may potentiate antitumor immune responses to immune checkpoint blockade therapy. This first-in-human, phase I/II dose escalation/expansion study assessed INCAGN01876, a humanized GITR-targeting agonistic mAb, for advanced solid tumors (NCT02697591).

**Patients and Methods:**

Dose was escalated by 0.03 to 20 mg/kg every 2 weeks; flat doses of 400 mg every 4 weeks and 300 mg every 2 weeks were also evaluated. The primary objective was safety/tolerability; secondary objectives were pharmacokinetics and preliminary efficacy; and exploratory objectives were immunogenicity, GITR occupancy, and immune biomarker assessment.

**Results:**

Among 100 patients enrolled [prior anti–PD-1/PD-L1 therapy, 47%; most common tumors: colorectal (19%) and melanoma (14%)], 2% had one dose-limiting toxicity (grade 4 hypoxia and grade 3 pleurisy). The MTD was not reached. Treatment-related adverse events (TRAE) occurred in 69% of patients, most frequently fatigue (17%) and pruritus (14%); 10% had grade ≥3 TRAEs, most commonly fatigue (3%); and 23% reported immune-related adverse events, most frequently generalized pruritus and generalized rash (7% each). Doses ≥5 mg/kg every 2 weeks resulted in full receptor occupancy at trough. INCAGN01876 elicited changes in immune parameters in some patients, including variable peripheral regulatory T-cell depletion and cytokine upregulation. Two patients achieved confirmed partial responses: one with appendiceal mucinous carcinoma and another with melanoma previously treated with pembrolizumab and glembatumumab; 36% of patients had disease control.

**Conclusions:**

INCAGN01876 was generally well tolerated; fatigue was the most frequent TRAE. INCAGN01876 elicited transient and variable regulatory T-cell depletion and limited antitumor activity. Future studies will explore combinatorial approaches.

**Translational Relevance**

Glucocorticoid-induced tumor necrosis factor receptor–related protein agonism in T cells may potentiate antitumor immunity and immune checkpoint blockade. This first-in-human, phase I/II dose escalation/expansion study assessed INCAGN01876, a humanized glucocorticoid-induced tumor necrosis factor receptor–related protein-targeting agonistic antibody, for advanced solid tumors. INCAGN01876 was well tolerated, elicited transient and variable immune responses, and was associated with limited antitumor activity. Future studies to assess INCAGN01876 in combination treatment regimens are warranted.

## Introduction

The immune system is designed to exert antigenic responses that are elegantly tuned to target pathogenic rather than host antigens via tight regulation. Such antigenic responses recognize, attack, and destroy tumor cells, thus mediating tumor immune surveillance (1). Coinhibitory and costimulatory immune receptors and their ligands play a critical role in regulating the immune response (2). Coinhibitory receptors include cytotoxic T-lymphocyte antigen 4 expressed in regulatory T cells (Treg), effector CD4^+^ T cells, and activated cytotoxic T cells; PD-1, mainly expressed on activated T cells, as well as NK cells, myeloid cells, neutrophils, and antigen-presenting cells [including B cells, monocytes, and dendritic cells (DC)]; and PD-L1 expressed in multiple cell types, including macrophages and activated DCs. Costimulatory receptors include those belonging to the CD28 family and the tumor necrosis factor receptor (TNFR) superfamily, such as OX40 and glucocorticoid-induced TNFR-related protein (GITR). mAbs that specifically bind to coinhibitory immune receptors and their ligands—subsequently blocking receptor–ligand interactions and consequently their downstream signaling—have been developed as immunotherapies in several tumor settings (3–7). Despite the demonstrated clinical efficacy of anti–PD-1/PD-L1 therapies for advanced solid tumors, the majority of patients still experience disease progression or become treatment-resistant (8).

Preclinical and clinical evidence has provided a rationale for evaluating anticancer agents that target costimulatory receptors belonging to the TNFR superfamily (9, 10). GITR is a TNFR superfamily member and is activated by the cognate ligand, GITRL (11). GITR is expressed on activated Tregs, effector T cells, transitional memory T cells, and NK cells (12–14). In humans, GITRL expression has been identified on activated plasmacytoid DC precursors and is thought to have a costimulatory role in NK cell activation (14). In mice, GITRL is constitutively expressed by freshly isolated B cells, as well as macrophages, DCs, and endothelial cells (14, 15), and its expression on DCs was found to be augmented in the tumor microenvironment (16). During T-cell priming, antigenic peptide-loaded MHC molecules are displayed on the surface of antigen-presenting cells in which they are recognized by T cells via the T-cell receptor (TCR; refs. 17, 18). TCR-mediated signaling leads to GITR upregulation in both CD4^+^ and CD8^+^ T cells (12, 13, 19, 20). In this context, GITR is an important costimulatory molecule in the activation of these T cells (12), leading to enhanced T-cell proliferation, survival, and an increased cytokine-mediated inflammatory response (21–24). The costimulatory activity of GITR in enhancing T-cell proliferation and survival signaling is mediated by the NF-κB pathway, which can prolong T-cell survival as a response to weak, rather than strong, TCR signals (10, 25–27). GITR is also expressed on Tregs that infiltrate the tumor microenvironment; notably, these tumor-infiltrating Tregs contribute to suppressing antitumor immune responses (28). However, costimulation by GITR during T-cell activation can inhibit this immune suppression. In tumors, this has been attributed to Treg cell differentiation and a potentially transient loss of *FoxP3* (29). GITR stimulation thereby acts by protecting effector T cells from Treg-mediated inhibition, as well as direct inhibition of Treg suppressive action (30). Modulation of the GITR costimulatory pathway may, therefore, provide a therapeutic strategy for targeting both effector T cells and Tregs in the tumor microenvironment, with the potential to increase T-cell responsiveness against relatively weak antigens, such as those expressed by tumor cells.

Agonist GITR mAbs have been shown to destabilize and deplete Tregs via mechanisms involving T-cell lineage changes and antibody-dependent cell-mediated cytotoxicity (ADCC), respectively (10, 31). Agonistic binding of the anti-mouse GITR mAb, DTA-1, to GITR on Tregs has been shown to result in lineage instability and conversion to nonregulatory T cells in mouse tumor models (10, 32). In this context, the divalent nature of the GITR mAbs confers an ability to mimic GITRL and cluster and activate GITR (33). In addition, GITR engagement with mAbs that are highly selective for activating Fc-gamma receptors (FcγR) has been shown to deplete Tregs via FcγR- and antibody-dependent cell-mediated phagocytosis or ADCC, which is associated with significant antitumor activity (31, 34). Therefore, targeting GITR may enhance antitumor immunity by increasing effector T-cell function and inhibiting the immunosuppressive effects of Tregs.

INCAGN01876 is a humanized agonistic IgG1-κ mAb that selectively binds to the extracellular domain of GITR (35, 36). Preclinical findings indicate that the agonistic GITR activity of INCAGN01876 may elicit antitumor activity by two mechanisms: (i) costimulatory agonistic engagement of GITR to enhance effector T-cell activity and (ii) coengagement of activating FcγRs to selectively deplete or destabilize immunosuppressive Tregs within the tumor microenvironment (35). Based on these preclinical lines of evidence supporting GITR as a potential antitumor therapeutic target, this first-in-human, phase I/II study assessed the safety, tolerability, pharmacokinetics (PK), pharmacodynamics (PD), and preliminary efficacy of INCAGN01876 in patients with locally advanced or metastatic solid tumors. The final study results are presented herein.

## Patients and Methods

### Study design

This was an open-label, nonrandomized, two-part, phase I/II, dose-escalation, dose-expansion study of INCAGN01876 for advanced or metastatic solid tumors (Supplementary Fig. S1; clinicaltrials.gov ID: NCT02697591). Part 1 (dose escalation) aimed to determine the pharmacologically active dose (PAD) and/or the MTD of INCAGN01876, the optimal dosing schedule, and the maximum number of tolerated doses (MNTD; see Supplementary Methods S1 for details). The PAD was defined as the dose providing the maximal biochemical effect or an increase in immune activity biomarkers. The starting dose was 0.03 mg/kg every 2 weeks; the dose was escalated using a standard 3 + 3 design with prespecified dose levels of 0.1, 0.3, 1, 3, 5, 10, and 20 mg/kg every 2 weeks. Two *post hoc* flat doses of 400 mg every 4 weeks and 300 mg every 2 weeks were also assessed. Patients received INCAGN01876 as a 30-minute intravenous infusion on day 1 of each 2- and 4-week cycle. Patients continued treatment as long as they were deriving clinical benefit and did not experience unacceptable toxicity or withdraw consent. Patients were assigned to up to 26 × 14-day treatment cycles, up to 17 × 21-day treatment cycles, or up to 13 × 28-day treatment cycles, with an expected maximum treatment duration of 14 months.

Part 2 (dose expansion) was aimed at further evaluating the safety, tolerability, preliminary efficacy, PK, and pharmacologic activity of the recommended phase II dose (RP2D) of INCAGN01876, using the administration schedule determined in part 1. Individual cohorts recruited patients with select tumor types responsive to PD-1 and cytotoxic T-lymphocyte antigen 4 [melanoma, non–small cell lung cancer (NSCLC); ref. 37] or tumor types shown to have tumor-infiltrating lymphocytes with elevated GITR expression [adenocarcinoma of the endometrium, melanoma, NSCLC, and renal cell cancer (RCC); Supplementary Methods S1].

The study was performed in compliance with the International Conference on Harmonization Good Clinical Practice guidelines, the Declaration of Helsinki, and applicable local regulations. The protocol was approved by the respective Institutional Review Boards of participating institutions. Written informed consent was obtained from all patients before study-related procedures were performed.

### Patients

Eligible patients were aged 18 years or older with locally advanced or metastatic solid tumors (part 1) or advanced/metastatic endometrial adenocarcinoma (documented microsatellite instability status), melanoma, NSCLC (squamous or nonsquamous), or histologically confirmed RCC (part 2). Patients had disease progression on prior therapies known to confer clinical benefit or were intolerant to prior treatment (including patient refusal of standard treatment). They had an Eastern Cooperative Oncology Group performance status of 0 or 1, laboratory and medical history parameters within protocol-defined ranges and commensurate with intact organ function, and had recovered from the toxic effects of prior therapy (including prior immunotherapy) to grade ≤1 severity.

Patients were excluded for prior receipt of the following treatments before the first dose: blood transfusion and colony-stimulating factors <14 days; chemotherapy, targeted small-molecule therapy, or radiotherapy ≤14 days; immunotherapy ≤42 days (part 1 only); immunotherapy or persistence of active cellular therapy ≤28 days (part 2 only); prior anticancer mAb therapy ≤28 days (except for denosumab); immunosuppressive-based treatment for any reason ≤7 days; and all other investigational study drugs or devices ≤28 days or five half-lives (whichever was longer).

### Objectives and assessments

The primary objective was to evaluate safety, tolerability, dose-limiting toxicities (DLT; Supplementary Table S1) and to define a PAD/MTD associated with INCAGN01876. Secondary objectives included the evaluation of PK and preliminary efficacy of INCAGN01876. Exploratory objectives included the assessment of INCAGN01876 immunogenicity, the association between INCAGN01876 PK and GITR receptor occupancy, biomarkers of INCAGN01876 pharmacologic activity, and the effects of INCAGN01876 on immune biomarkers.

Safety was assessed from physical examination, vital signs, laboratory assessments, 12-lead electrocardiograms, and treatment-emergent adverse events (TEAE) coded per Medical Dictionary for Regulatory Activities (RRID:SCR_003751). TEAE severity was graded 1 to 4, using NCI Common Terminology Criteria for Adverse Events version 4.03. Immune-related adverse events (irAE) were investigator-identified and defined as adverse events consistent with an immune phenomenon associated with drug exposure after all other etiologies had been eliminated.

Efficacy was evaluated by assessment of objective response, duration of response, and disease control rate (complete response + partial response + stable disease). Radiologic imaging assessments (CT/MRI) were performed at week 8 and then every 8 weeks for the first 12 months and then every 12 weeks thereafter. Response was assessed using RECIST (RRID:SCR_026435) v1.1 and modified RECIST v1.1 (38), which is an adapted version of RECIST v1.1 that considers the unique tumor response characteristics seen with immunotherapy.

### PK and antidrug antibody assessments

Blood serum samples were collected before the dose and at various time points during the study (Supplementary Methods S1). Patient serum samples were analyzed for PK using a validated ELISA, and a validated electrochemiluminescence method was used to detect antidrug antibodies (ADA) to INCAGN01876 in serum.

INCAGN01876 serum concentration data were analyzed based on a standard noncompartmental PK model using Phoenix WinNonlin v8.3.2 (RRID:SCR_024504) and graphed using R v3.6.1 (The R Project for Statistical Computing; RRID:SCR_001905). PK assessments included only ADA-negative patients. An accumulation ratio was computed as the ratio of the area under the single-dose plasma or serum concentration–time curve (AUC) over the dosing interval (i.e., AUC_336_) on cycle 1 day 1 (C1D1) compared with C6D1. The dose proportionality of INCAGN01876 serum exposure was evaluated using a power function regression. C_max_ and AUC were evaluated using a power model, for example, AUC = α∙(dose β) or, equivalently, log(AUC) = log(α) + β∙log(dose), in which linear dose proportionality was accepted if β was not significantly different from unity.

### GITR receptor occupancy

GITR receptor occupancy was assessed using plasma samples from patients enrolled in the dose escalation cohorts (three to four patients per cohort; see Supplementary Methods S1 for details). The relationship between INCAGN01876 exposure and GITR receptor occupancy was determined using maximal effect modeling, performed in R v3.6.1 using the nls function.

### Plasma protein analyses

To extend PD evaluations of INCAGN01876, immune- and nonimmune-related plasma protein expression was determined using a multiplex Proximity Extension Assay developed and performed by Olink Proteomics. The effect of INCAGN01876 treatment on biomarkers was assessed by comparing baseline (C1D1) values to on-treatment values (C1D2, C1D7, and/or C2D1).

### Circulating immune cell analysis

Circulating immune cells were analyzed in peripheral blood from patients with available samples by multicolor flow cytometry using a panel comprising markers for memory T cells, Tregs, and T-cell activation/exhaustion (Supplementary Table S2). To complement these studies, circulating immune cell analyses were also performed on peripheral blood mononuclear cells from a patient subpopulation enrolled at Memorial Sloan Kettering Cancer Center (MSKCC) by FACS using a 15-color panel (Supplementary Table S3), as described by Zappasodi and colleagues (39). Frequencies of immune cells were monitored at screening and at various time points during treatment (Supplementary Methods S1).

Tumor infiltration by T cells was assessed in pretreatment (at screening) and posttreatment tumor biopsy samples using IHC. Cell densities were determined in tumor versus nontumor regions using pancytokeratin (PanCK)/S100 as a segmentation marker. Paired biopsy samples were first analyzed by a nine-color multiplex IHC panel (Supplementary Table S4) using the MultiOmyx platform (NeoGenomics). Tumor cells were stained using S100 for melanomas and PanCK for other tumor types. A second analysis was performed using a seven-marker immunofluorescence panel at MSKCC; paired biopsy samples from seven patients were analyzed using a seven-plex Vectra immunofluorescence panel (Vectra Polaris Automated Quantitative Pathology Imaging System; RRID:SCR_025508; Supplementary Table S5). All posttreatment biopsies were performed between the start of cycle 3 and the end of cycle 4 (i.e., approximately between week 5 and week 9 after starting treatment for patients on 14-day cycles and between week 9 and week 16 for those on 4-week cycles).

### Statistical analysis

In part 1, the sample size was the total number of patients enrolled in dose levels to evaluate the MTD and/or PAD. In part 2, the sample size of each tumor cohort was guided by a Simon two-stage design (Supplementary Methods S1). The full analysis set included all enrolled patients (parts 1 and 2) who had received at least one dose of INCAGN01876. The PK, ADA, PD, and biopsy evaluable populations included patients who had received at least one dose of INCAGN01876 and had at least one postdose PK, PD, ADA, and biopsy sample collected and analyzed. For this analysis, data from dose cohorts 0.03, 0.1, 0.3, and 1 mg/kg every 2 weeks (*n* = 15) were pooled. Descriptive statistics were used to summarize demographics, disease characteristics, PK, PD, and response.

### Data availability

Incyte Corporation is committed to data sharing that advances science and medicine while protecting patient privacy. Qualified external scientific researchers may request anonymized datasets owned by Incyte for the purpose of conducting legitimate scientific research by submitting a request via email to datasharing@incyte.com.

## Results

### Patients

**Figure 1.**
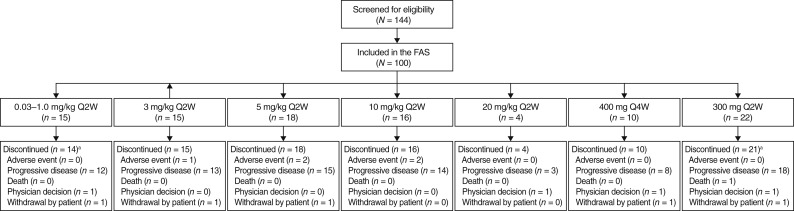
Patient disposition. ^a^Two patients completed treatment [0.3 mg/kg every 2 weeks (Q2W) and 300 mg Q2W, respectively]. The death in the 300-mg Q2W cohort was coded as dyspnea secondary to disease progression. Reasons for patient ineligibility included meeting the following exclusion criteria: (i) laboratory and medical history parameters not within the protocol-defined range (39%; 17/44 patients); (ii) any condition that would, in the investigator’s judgment, interfere with full participation in the study (including administration of the study drug and attending required study visits), pose a significant risk to the subject, or interfere with the interpretation of study data (16%; 7/44 patients); (iii) known active central nervous system metastases and/or carcinomatous meningitis (14%; 6/44 patients); and (iv) evidence of hepatitis B virus (HBV) or hepatitis C virus (HCV) infection or risk of reactivation (positive testing for HBV DNA and/or HCV RNA; 11%; 5/44 patients) and not meeting the following inclusion criteria: willingness to provide a written informed consent form (7%; 3/44 patients) and having Eastern Cooperative Oncology Group performance status of 0 or 1 (5%; 2/44 patients). FAS, full analysis set.

Among 144 patients screened for eligibility, 100 were enrolled and included in the full analysis set at seven study sites in the United States between June 20, 2016, and December 16, 2019 (Fig. 1). Two patients (2%, 0.3 mg/kg every 2 weeks and 300 mg every 2 weeks, *n* = 1 each) completed their assigned maximum number of treatment cycles (26 × 14-day cycles), and 98 (98%) discontinued treatment, most commonly owing to progressive disease [*n* = 83 (83%)], as well as adverse events [*n* = 5 (5%)], consent withdrawal [*n* = 5 (5%)], physician decision [*n* = 4 (4%)], and death [*n* = 1 (1%); dyspnea, secondary to disease progression]. Among enrolled patients, the median age was 62 years (range, 27–87); most were male (58%) and White (82%). The most common tumors were colorectal [*n* = 19 (19%)], melanoma [*n* = 14 (14%)], and NSCLC [*n* = 12 (12%)]; 42% of patients had gastrointestinal tumors. All 100 patients (100%) had received prior systemic therapy; 49 patients (49%) had received prior immunotherapy, almost all of whom had received prior anti–PD-1/PD-L1 therapy (*n* = 47; Table 1). Baseline characteristics of the patient subgroups whose samples were used in separate FACS and IHC analyses, including those enrolled at MSKCC, were generally consistent with those of the overall population (Supplementary Tables S6 and S7).

**Table 1.** Baseline demographics and characteristics.

Characteristic	Treatment group							
	0.03–1 mg/kg Q2W(*n* = 15)	3 mg/kg Q2W(*n* = 15)	5 mg/kg Q2W(*n* = 18)	10 mg/kg Q2W(*n* = 16)	20 mg/kg Q2W(*n* = 4)	400 mg Q4W(*n* = 10)	300 mg Q2W(*n* = 22)	Total(*N =* 100)
Median age, years (range)	59 (27–87)	64 (42–82)	58 (27–76)	61.5 (43–83)	62 (40–74)	55.5 (38–76)	69 (35–83)	62 (27–87)
>65 years	4 (26.7)	7 (46.7)	2 (11.1)	4 (25)	2 (50)	3 (30)	15 (68.2)	37 (37)
Sex, *n* (%)	​	​	​	​	​	​	​	​
Female	7 (46.7)	7 (46.7)	11 (61.1)	11 (68.8)	3 (75)	6 (60)	12 (54.5)	58 (58)
Male	8 (53.3)	8 (53.3)	7 (38.9)	5 (31.3)	1 (25)	4 (40)	10 (45.5)	42 (42)
Race, *n* (%)	​	​	​	​	​	​	​	​
White/Caucasian	10 (66.7)	13 (86.7)	15 (83.3)	12 (75)	4 (100)	7 (70)	21 (95.5)	82 (82)
Black/African American	3 (20)	2 (13.3)	1 (5.6)	2 (12.5)	0	0	1 (4.5)	9 (9)
Asian	1 (6.7)	0	0	2 (12.5)	0	2 (20)	0	5 (5)
Missing	1 (6.7)	0	2 (11.1)	0	0	1 (10)	0	4 (4)
ECOG performance status, *n* (%)	​	​	​	​	​	​	​	​
0	9 (60)	8 (53.3)	10 (55.6)	8 (50)	1 (25)	6 (60)	12 (54.5)	54 (54)
1	6 (40)	7 (46.7)	8 (44.4)	8 (50)	3 (75)	4 (40)	10 (45.5)	46 (46)
Tumor type^a^, *n* (%)	​	​	​	​	​	​	​	​
Colorectal cancer^b^	1 (6.7)	2 (13.3)	6 (33.3)	7 (43.8)	1 (25)	2 (20)	0	19 (19)
Melanoma	1 (6.7)	2 (13.3)	1 (5.6)	0	0	1 (10)	9 (40.9)	14 (14)
NSCLC	1 (6.7)	1 (6.7)	0	1 (6.3)	0	0	9 (40.9)	12 (12)
Renal cell carcinoma	0	1 (6.7)	1 (5.6)	1 (6.3)	0	0	4 (18.2)	7 (7)
Breast cancer	1 (6.7)	1 (6.7)	3 (16.7)	1 (6.3)	0	0	0	6 (6)
Pancreatic cancer^c^	2 (13.3)	1 (6.7)	0	2 (12.5)	0	1 (10)	0	6 (6)
Biliary tract cancer^d^	0	1 (6.7)	1 (5.6)	0	2 (50)	1 (10)	0	5 (5)
Prior platinum chemotherapy	12 (80)	9 (60)	13 (72.2)	13 (81.3)	3 (75)	7 (70)	10 (45.5)	67 (67)
Prior radiotherapy	11 (73.3)	8 (53.3)	10 (55.6)	6 (37.5)	2 (50)	4 (40)	11 (50)	52 (52)
Prior surgery	13 (86.7)	13 (86.7)	16 (88.9)	14 (87.5)	4 (100)	6 (60)	19 (86.4)	85 (85)
Prior immunotherapy	5 (33.3)	8 (53.3)	5 (27.8)	5 (31.3)	0	4 (40)	22 (100)	49 (49)
Anti–PD-1/PD-L1 therapy	4 (26.7)	8 (53.3)	5 (27.8)	4 (25)	0	4 (40)	22 (100)	47 (47)

Abbreviations: ECOG, Eastern Cooperative Oncology Group; Q2W, every 2 weeks; Q4W, every 4 weeks.

a Tumors occurring in ≥5 patients at the screening visit; other tumor types included sarcoma (*n* = 4), neuroendocrine cancer (*n* = 3), urothelial tract/bladder cancer (*n* = 3), gastric cancer (*n* = 2), head and neck cancer (*n* = 2), ovarian cancer (*n* = 2), adrenal cancer (*n* = 1), anal cancer (*n* = 1), appendiceal carcinoma (*n* = 1), appendiceal mucinous (*n* = 1), chondrosarcoma (*n =* 1), endometrial cancer (*n =* 1), hepatocellular carcinoma (*n =* 1), paranasal sinus cancer (*n =* 1), prostate cancer (*n =* 1), small cell lung cancer (*n =* 1), small intestine cancer (*n =* 1), thymic cancer (*n =* 2), thyroid cancer (*n =* 1), and uterine cancer (*n =* 1).

b Colorectal cancer includes colorectal (*n =* 16), cecum and ascending colon (*n =* 1), colon (*n =* 1), and rectal (*n =* 1) cancers.

c Pancreatic cancer includes mucinous (*n =* 3), neuroendocrine (*n =* 2), and endocrine pancreatic (*n =* 1) cancers.

d Biliary tract cancer includes cholangiocarcinoma (*n =* 4) and gallbladder (*n =* 1) cancers.

### Exposure and dose escalation

Patients in the full analysis set received predefined INCAGN01876 doses of 0.03 mg/kg every 2 weeks (*n* = 4), 0.1 mg/kg every 2 weeks (*n* = 4), 0.3 mg/kg every 2 weeks (*n* = 4), 1 mg/kg every 2 weeks (*n* = 3), 3 mg/kg every 2 weeks (*n* = 15), 5 mg/kg every 2 weeks (*n* = 18), 10 mg/kg every 2 weeks (*n* = 16), and 20 mg/kg every 2 weeks (*n* = 4) and *post hoc* doses of 400 mg every 4 weeks (*n* = 10) and 300 mg every 2 weeks (*n* = 22). Patients received a median of four (range, 1–26) INCAGN01876 infusions. The median (range) number of infusions ranged from 3 (2–6) and 3 (2–10) for the 0.03 and 1 mg/kg every 2 weeks cohorts to 9.5 (3–26) for the 0.3 mg/kg every 2 weeks cohort; they were 3.5 (2–9) and 4.5 (1–26) for the 400 mg every 4 weeks and 300 mg every 2 weeks cohorts, respectively. Overall, the median duration of treatment was 43.5 days (range, 1–367).

### Safety

Two patients (2%) had one DLT each: One patient in the 3 mg/kg every 2 weeks cohort had a grade 4 DLT of hypoxia, and one in the 5 mg/kg every 2 weeks cohort had a grade 3 DLT of pleurisy, both occurring after the second INCAGN01876 infusion. The MTD and MNTD were not reached (Supplementary Results).

Among patients enrolled, all (100%) reported at least one TEAE, most frequently (≥20% of patients) fatigue [*n* = 40 (40%)], abdominal pain [*n* = 26 (26%)], dyspnea [*n* = 26 (26%)], nausea [*n* = 25 (25%)], pyrexia [*n* = 21 (21%)], and decreased appetite [*n* = 20 (20%); Table 2]. Grade ≥3 TEAEs occurred in 57 patients (57%), most frequently (>5% of patients) malignant neoplasm progression [*n* = 10 (10%)], fatigue [*n* = 8 (8%)], dyspnea [*n* = 7 (7%)], and abdominal pain [*n* = 7 (7%)]. Treatment-related adverse events (TRAE) occurred in 69 patients (69%), most frequently (≥10% of patients) fatigue [*n* = 17 (17%)] and pruritus [*n* = 14 (14%); Supplementary Table S8]. Among patients with pruritus or rash-related TEAEs, no clear trend was observed between the timing of INCAGN01876 infusions and the onset of events. Symptoms were managed with corticosteroids in four patients (for seven episodes) with pruritus, four patients with generalized pruritus (all patients *n* = 1), four patients (for five episodes) with generalized rash, and one patient with maculopapular rash (Supplementary Table S9). Grade ≥3 TRAEs occurred in 10 patients (10%), most frequently (>1 patient) fatigue [*n* = 3 (3%)], anemia [*n* = 2 (2%)], and dyspnea [*n* = 2 (2%)]. No clear trend was seen between the timing of INCAGN01876 infusions and the onset of events (Supplementary Table S10). Serious TEAEs were reported in 43 patients (43%), most commonly (≥3 patients) malignant neoplasm progression [*n* = 10 (10%)], abdominal pain [*n* = 5 (5%)], dyspnea [*n* = 4 (4%)], dehydration, and sepsis [*n* = 3 (3%) each]. Fatal TEAEs occurred in 17 patients (17%), including malignant neoplasm progression [*n* = 9 (9%)], brain edema, cardiac arrest, cardiorespiratory arrest, cardiogenic shock, dyspnea secondary to progressive disease, respiratory failure, sepsis, septic shock, and thrombotic stroke [*n* = 1 (1%) each; Supplementary Table S11]. None of the fatal TEAEs were deemed treatment-related (per investigators).

**Table 2.** TEAEs^a^.

Adverse event, *n* (%)^b^	Treatment group															
	0.03–1 mg/kg Q2W(*n* = 15)		3 mg/kg Q2W(*n* = 15)		5 mg/kg Q2W(*n* = 18)		10 mg/kg Q2W(*n* = 16)		20 mg/kg Q2W(*n* = 4)		400 mg Q4W(*n* = 10)		300 mg Q2W(*n* = 22)		Total^b^(*N* = 100)	
	Any Gr	Gr ≥ 3	Any Gr	Gr ≥ 3	Any Gr	Gr ≥ 3	Any Gr	Gr ≥ 3	Any Gr	Gr ≥ 3	Any Gr	Gr ≥ 3	Any Gr	Gr ≥ 3	Any Gr	Gr ≥ 3
Fatigue	2 (13.3)	0	7 (46.7)	1 (6.7)	6 (33.3)	1 (5.6)	7 (43.8)	0	2 (50)	1 (25)	5 (50)	0	11 (50)	5 (22.7)	40 (40)	8 (8)
Abdominal pain^c^	4 (26.7)	0	5 (33.3)	1 (6.7)	5 (27.8)	2 (11.1)	10 (62.5)	0	3 (75)	2 (50)	3 (30)	1 (10)	2 (9.1)	1 (4.5)	31 (31)	7 (7)
Dyspnea	2 (13.3)	0	7 (46.7)	3 (20)	4 (22.2)	1 (5.6)	3 (18.8)	1 (6.3)	1 (25)	0	3 (30)	0	6 (27.3)	2 (9.1)	26 (26)	7 (7)
Nausea	2 (13.3)	0	3 (20)	0	5 (27.8)	0	6 (37.5)	0	1 (25)	0	2 (20)	0	6 (27.3)	1 (4.5)	25 (25)	1 (1)
Pyrexia	5 (33.3)	0	2 (13.3)	0	3 (16.7)	0	5 (31.3)	0	0	0	2 (20)	0	4 (18.2)	0	21 (21)	0
Decreased appetite	2 (13.3)	1 (6.7)	4 (26.7)	0	2 (11.1)	0	3 (18.8)	0	2 (50)	0	3 (30)	0	4 (18.2)	0	20 (20)	1 (1)
Vomiting	3 (20)	0	3 (20)	0	3 (16.7)	0	5 (31.3)	0	2 (50)	0	1 (10)	0	2 (9.1)	0	19 (19)	0
Anemia	2 (13.3)	1 (6.7)	2 (13.3)	1 (6.7)	4 (22.2)	1 (5.6)	3 (18.8)	1 (6.3)	0	0	1 (10)	0	5 (22.7)	1 (4.5)	17 (17)	5 (5)
Diarrhea	2 (13.3)	0	4 (26.7)	1 (6.7)	4 (22.2)	1 (5.6)	2 (12.5)	0	1 (25)	0	0	0	4 (18.2)	1 (4.5)	17 (17)	3 (3)
Edema peripheral	1 (6.7)	0	3 (20)	0	5 (27.8)	0	0	0	1 (25)	0	1 (10)	0	6 (27.3)	2 (9.1)	17 (17)	2 (2)
Pruritus	1 (6.7)	0	5 (33.3)	0	5 (27.8)	0	3 (18.8)	0	0	0	1 (10)	1 (10)	2 (9.1)	0	17 (17)	1 (1)
Cough	2 (13.3)	0	5 (33.3)	1 (6.7)	1 (5.6)	0	1 (6.3)	0	0	0	0	0	5 (22.7)	0	14 (14)	1 (1)
Dehydration	1 (6.7)	0	2 (13.3)	1 (6.7)	0	0	2 (12.5)	1 (6.3)	1 (25)	0	2 (20)	0	4 (18.2)	3 (13.6)	12 (12)	5 (5)
Headache	4 (26.7)	0	2 (13.3)	0	4 (22.2)	0	0	0	0	0	0	0	2 (9.1)	0	12 (12)	0
Back pain	5 (33.3)	2 (13.3)	1 (6.7)	0	0	0	2 (12.5)	1 (6.3)	0	0	1 (10)	0	2 (9.1)	0	11 (11)	3 (3)
Constipation	0	0	1 (6.7)	0	5 (27.8)	0	1 (6.3)	0	1 (25)	0	0	0	3 (13.6)	1 (4.5)	11 (11)	1 (1)
Blood ALP increased	1 (6.7)	0	1 (6.7)	1 (6.7)	2 (11.1)	1 (5.6)	3 (18.8)	1 (6.3)	1 (25)	1 (25)	0	0	2 (9.1)	0	10 (10)	4 (4)

Abbreviations: ALP, alkaline phosphatase; Gr, grade.

a TEAEs reported in ≥10% of patients by MedDRA preferred term in decreasing order of frequency (total column).

b Ten patients (10%) had a TEAE of malignant neoplasm progression, which was classified as disease under study.

c Includes preferred terms of abdominal pain, abdominal pain upper, and abdominal pain lower.

Five patients (5%) discontinued treatment owing to TEAEs, including acute myocardial infarction, brain edema, cardiorespiratory arrest, hypersensitivity, large intestinal obstruction, and respiratory failure [*n* = 1 (1%) each; Supplementary Table S12]. TEAEs led to infusion interruption in 20 patients (20%), including 19 with dose delays and one with interruption due to hypersensitivity. The most frequently reported (≥2 patients) TEAEs leading to infusion interruption were fatigue [*n* = 3 (3%)] and anemia, diarrhea, and malaise [*n* = 2 (2%) each]. No patient had a TEAE leading to dose reduction (Supplementary Table S13).

A total of 23 patients (23%) had at least one investigator-identified irAE, most frequently (≥2 patients) generalized pruritus and generalized rash [*n* = 7 (7%) each], pruritus [*n* = 5 (5%)], fatigue and rash [*n* = 3 (3%) each], and dyspnea [*n* = 2 (2%)]. Grade ≥3 irAEs occurred in five patients (5%), including dermatitis bullous, diarrhea, dyspnea, fatigue, pleurisy, and pruritus [*n* = 1 (1%) each]. One patient receiving 5 mg/kg every 2 weeks had an investigator-identified grade 2 irAE of infusion-related hypersensitivity associated with hypotension, rash, and abdominal pain. Upon retrospective review, an additional irAE of grade 2 infusion-related hypersensitivity was identified in the same patient by the sponsor, which was associated with chills, dizziness, hypotension, nausea, diarrhea, light-headedness, pain, and rigors, and led to treatment discontinuation. Both events were reported shortly after the patient tested ADA-positive at the beginning of cycle 3. A second patient receiving 5 mg/kg every 2 weeks had a grade 2 sponsor-assessed episode of hypersensitivity leading to dose interruption; this patient was ADA-negative.

### PK of INCAGN01876 and incidence of ADAs

**Figure 2.**
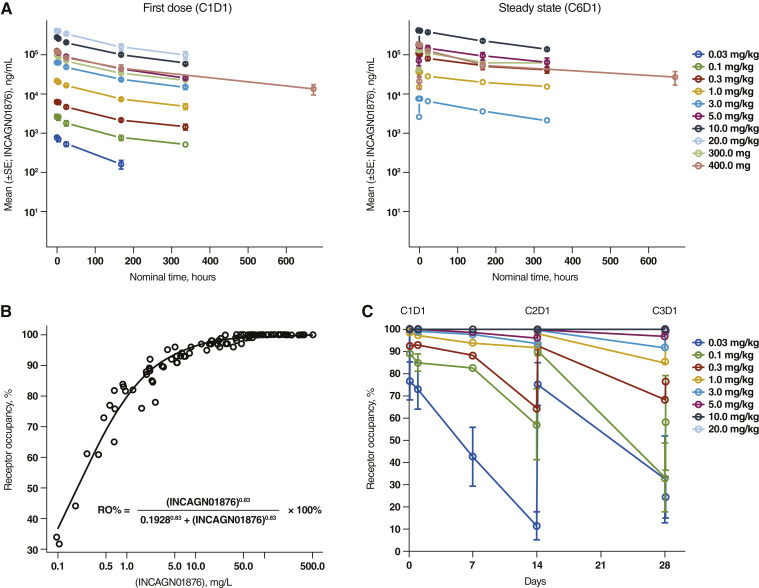
**A,** INCAGN01876 mean (±SE) concentration–time profiles after the first dose and at steady state. **B,** Relationship between serum concentrations of INCAGN01876 and GITR receptor occupancy. **C,** Percentage of INCAGN01876 receptor occupancy vs. time. The average receptor occupancy per cohort is depicted (± SD). PK analysis includes patients with ADA-negative status at the respective visit; first visit/steady state: 0.03 mg/kg, *n* = 4/*n* = 0; 0.1 mg/kg, *n* = 4/*n* = 0; 0.3 mg/kg, *n* = 3/*n* = 1; 1 mg/kg, *n* = 3/*n* = 1; 3 mg/kg, *n* = 15/*n* = 5; 5 mg/kg, *n* = 16/*n* = 5; 10 mg/kg, *n* = 14/*n* = 4; 20 mg/kg, *n* = 3/*n* = 0; 300 mg, *n* = 21*/n* = 7; and 400 mg, *n* = 9/*n* = 3. RO, receptor occupancy.

Mean INCAGN01876 serum concentration–time profiles by dosing regimen on C1D1 and C6D1 for all ADA-negative patients are shown in Fig. 2A. Based on predose samples, steady-state serum concentrations were generally reached at cycle 6, approximately 2,000 hours after the first dose (Supplementary Fig. S2). Across all doses at steady state (cycle 6), median t_max_ was 0.667 hours, and geometric mean half-life was 246 hours (Supplementary Table S14). The geometric mean accumulation ratio was 1.84-fold (range, 1.17-fold to 2.68-fold). For doses ≥1 mg/kg, dose proportionality was achieved for both AUC_inf_ and C_max_ [slope, 1.06; 95% confidence interval (CI), 0.942–1.17, and slope, 1.02; 95% CI, 0.938–1.10, respectively].

The incidence of ADA was high at doses ≤0.3 mg/kg every 2 weeks [83.3% (10/12 patients); Supplementary Table S15] while being much lower at doses ≥1 mg/kg every 2 weeks [5.7% (5/88 patients)], potentially explaining the observed dose proportionality within this dose range. None of the 41 patients receiving doses of 3 mg/kg every 2 weeks, 10 mg/kg every 2 weeks, or 400 mg every 4 weeks had an occurrence of ADA. Of the 15 patients with confirmed ADA, one in the 20 mg/kg every 2 weeks cohort had ADA before the dose, and all other ADAs were treatment-emergent. In general, there was a dramatic reduction in INCAGN01876 exposure in ADA-positive patients compared with ADA-negative patients between cycle 1 to cycle 6 (Supplementary Fig. S3).

### GITR receptor occupancy

A direct relationship was observed between GITR receptor occupancy and INCAGN01876 concentration (Fig. 2B). Fitting these data to a sigmoidal E_max_ model yielded an EC_50_ of 0.1928 mg/L and a Hill coefficient of 0.83; the EC_90_ (i.e., 90% receptor occupancy) was 2.72 mg/L. All patients receiving doses ≥5 mg/kg every 2 weeks presented saturated receptor occupancy after infusion, with average receptor occupancy maintained above 90% at trough INCAGN01876 serum concentrations (Fig. 2C). INCAGN01876 administered at a dose of 1 mg/kg resulted in end-of-first-cycle (predose C2D1) serum concentrations above EC_90_ in three out of three patients, whereas a 0.3 mg/kg dose was associated with predose C2D1 serum concentrations below EC_90_ in three out of three patients.

### Plasma protein analysis

Plasma protein expression levels exhibiting a ≥1.5-fold increase from baseline upon INCAGN01876 treatment were considered significant (*P* < 0.05). CCL17 (*P* = 0.031), CD70 (*P* = 0.038), CLEC4G (*P* = 0.031), BIRC2 (*P* = 0.045), MK (*P* = 0.049), and BMP-4 (*P* = 0.022) showed significant upregulation; upregulation was also observed for IFNγ, CXCL-9, and CXCL-10; however, this seemed to be nonsignificant. For most upregulated proteins, increases in expression peaked at C1D7. Among plasma proteins displaying significantly increased expression, CCL17, CD70, and CLEC4G were analyzed further due to their demonstrated roles in T-cell activity and function (40–42). Percentage changes in CCL17, CD70, and CLEC4G expression levels from C1D1 to various time points are shown in Supplementary Fig. S4A. The time dependence of the upregulation of these cytokines varied by dose (Supplementary Fig. S4B). For the chemokine CCL17, the 5 mg/kg dose group demonstrated the most robust upregulation; however, for CD70 and CLEC4G, the 3 and 10 mg/kg doses led to greater increases for the respective ligand proteins. Upregulation of IFNγ-inducible cytokines, such as CXCL9 and CXCL10, which are used as PD markers for immune checkpoint blockade activity (43), was not statistically significant (data on file).

### Circulating immune cell analysis

**Figure 3.**
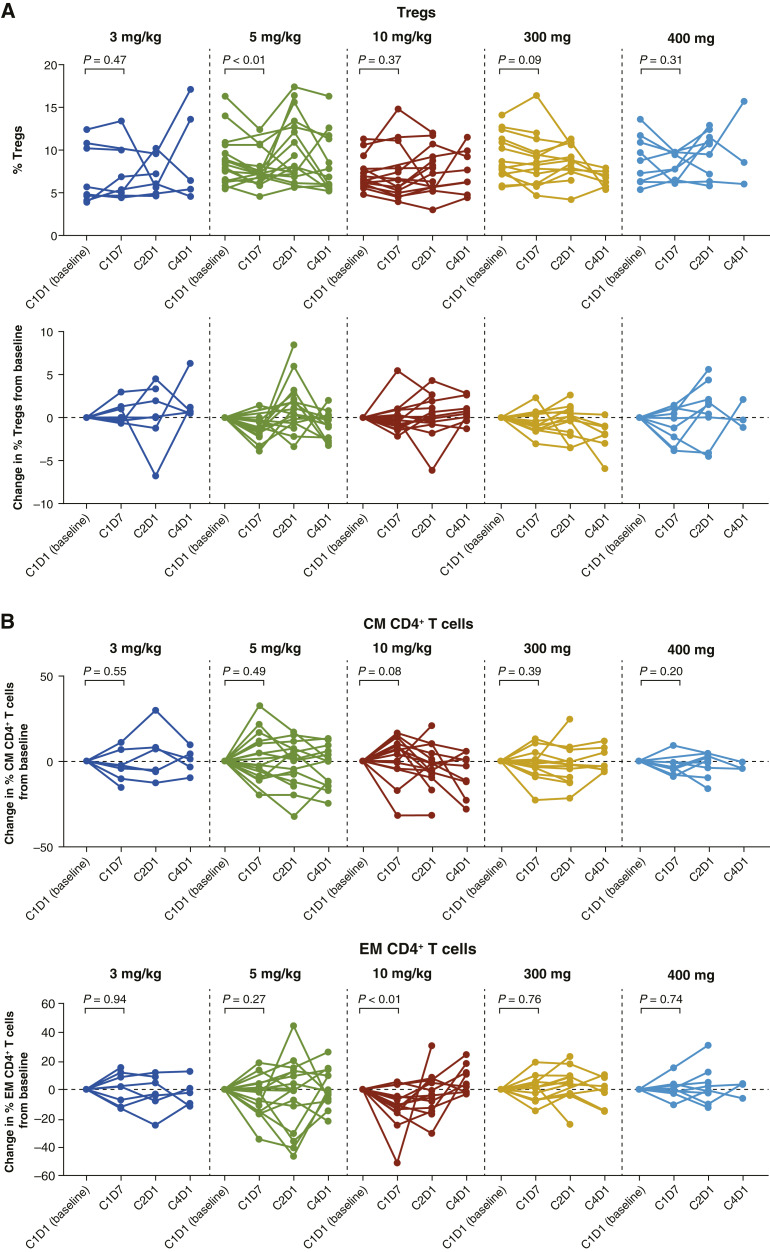
**A,** Frequencies of Tregs. **B,** Changes in central memory (CM) and EM CD4^+^ T cells.

Markers for T cells, Tregs, and T-cell activation/exhaustion were analyzed in peripheral blood samples from 64 patients by multicolor flow cytometry. No consistent longitudinal trends in absolute CD4^+^ and CD8^+^ T-cell counts were observed for any of the INCAGN01876 dose cohorts (data on file). The hypothesized mechanism of action of INCAGN01876 was to activate effector T cells or to inhibit, reprogram, and/or deplete Tregs (44). Supporting this hypothesis, the frequency of Tregs (CD3^+^ CD4^+^ CD25^+^ CD127^−^ FoxP3^+^) decreased significantly from baseline to C1D7 in the 5 mg/kg cohort (*P* < 0.01) and to a lesser (nonsignificant) degree in the 300 mg every 2 weeks cohort (*P* = 0.09; Fig. 3A) although similar decreases were not seen in the other cohorts. A moderate increase was observed across time points in central memory (CD45RO^+^CCR7^+^) CD4^+^ T cells (Fig. 3B), but not in CD8^+^ T cells (data on file). Significant decreases in effector memory (EM; CD45RO^+^CCR7^−^) CD4^+^ T cells (Fig. 3B) and CD8^+^ T cells (data on file) were observed in the 10 mg/kg cohorts at C1D7; the decrease in EM CD4^+^ T-cell frequencies for individual patients reached approximately 50% from baseline at this time point (Fig. 3B). Treg frequencies recovered to baseline by C2D1; no decrease was observed in either lower or higher dose groups. Expression of the HLA-DR T-cell activation marker on CD8^+^ T cells increased significantly at C4D1 in the 5 mg/kg cohort, and at C2D1 and C4D1 in the 300 mg flat dose cohort; CD38 expression showed similar trends (data on file). Ki67 expression in CD3^+^ T cells, a measure of T-cell proliferation, remained unchanged across all doses assessed (data on file).

GITR expression was analyzed in peripheral blood T cells from eight patients enrolled at MSKCC (Supplementary Table S3) by FACS using a panel that included an anti-GITR mAb with confirmed ability to bind GITR in the presence of INCAGN01876 (ebioAITR, Thermo Fisher Scientific; RRID:AB_2573785). The results demonstrated a greater reduction in GITR^+^ Tregs compared with total Tregs during treatment (Supplementary Fig. S5A and S5B). This observation is also consistent with the hypothesized mechanism of action of INCAGN01876. Of note, concomitant with the observed reduction in GITR^+^ Tregs, CD8^+^ Ki67^+^ T-cell levels increased during treatment in this subset of patients (Supplementary Fig. S5C), with similar changes in these cell populations occurring in tumors in the majority of patients analyzed (see below). Changes in GITR^+^ Tregs and CD8^+^ Ki67^+^ T-cell levels between baseline and C2D1 (selected as a time point when all patients had available samples) were examined and showed significant increases in CD8^+^ Ki67^+^ T-cell levels; a trend toward a reduction in GITR^+^ Tregs was also seen, but this did not reach significance (Supplementary Fig. S5D and S5E). However, these findings have not been verified in the overall study population. Gating strategy data and representative flow plots are shown in Supplementary Figs. S6 and S7.

### Tumor infiltration of T cells

Tumor infiltration of T cells was analyzed by IHC in paired biopsy samples from 9 out of 10 patients with available samples using a nine-color multiplex IHC panel (Supplementary Table S3). Increases in total and/or activated T cells in tumors were seen in seven of nine evaluable patients; two patients with melanoma did not show any increase in T-cell infiltration (Supplementary Fig. S8A–S8F). However, no consistent changes were observed in the CD8^+^/Treg ratio in this subset of patients. Tumor infiltration of various T-cell types was also assessed by seven-color multiplex immunofluorescence staining in paired screening and on-treatment biopsy samples from seven patients at MSKCC with available samples (Supplementary Table S2), using a panel that included a GITR marker. Tumor infiltration was numerically increased across a range of T-cell types for six of seven patients: Tregs (3/7 biopsy pairs), Ki67^+^ Tregs (3/7, of which 2 also displayed total Treg increases), CD8^+^ memory T cells (3/7), Ki67^+^ CD8^+^ proliferating T cells (4/7), CD4^+^ effector T cells (2/7), and GITR^+^ CD4^+^ effector T cells (2/7; Supplementary Fig S9A–S9G). GITR^+^ Tregs were decreased in all but one patient, where these cells remained quantitatively unchanged upon INCAGN01876 treatment, possibly due to the increase in total Tregs and their proliferation potential based on Ki67 expression in this patient. Although fixation and antigen retrieval should remove INCAGN01876 binding in tissue, it is not known if the GITR mAb clone used in this immunofluorescence analysis is blocked by INCAGN01876. Of note, in four of seven cases, CD8/Treg ratios increased, including in those displaying increased Treg frequency after INCAGN01876 treatment. This suggests that CD8^+^ T-cell increases could outcompete possible Treg increases upon treatment with INCAGN01876. Overall, these data suggest that INCAGN01876 treatment is associated with increased tumor infiltration of T cells, including some subsets of Tregs in certain cases. Representative IHC images are shown in Supplementary Fig. S10A and S10B.

### Efficacy

Two confirmed best overall responses of partial response were achieved (Supplementary Table S16): one in a patient with appendiceal mucinous carcinoma receiving 0.3 mg/kg every 2 weeks and one in a patient with melanoma receiving 300 mg every 2 weeks who had received prior treatment with pembrolizumab and glembatumumab. Neither patient was analyzed for immune correlates by FACS/IHC; neither was ADA-positive. The duration of partial response in the patient receiving 0.3 mg/kg every 2 weeks was 5.6 months; the partial response in the patient receiving 300 mg every 2 weeks was ongoing at the time of study termination (13.1 months after the initial response). Responses were associated with reductions in tumor size of 61% and 84%, respectively. Thirty-six patients (36%) had disease control; the median duration of disease control was 61 days (95% CI, 59–113).

## Discussion

This first-in-human study assessed the safety, tolerability, PK, preliminary efficacy, immunogenicity, and correlates of pharmacologic and immunologic activity of INCAGN01876 in patients with locally advanced or metastatic solid tumors. The results demonstrate that INCAGN01876 was generally well tolerated at doses ranging from 0.03 to 20 mg/kg every 2 weeks, 400 mg every 4 weeks, and 300 mg every 2 weeks, with two patients reporting DLTs and most irAEs (18/23, 78.3%) being of grade 1/2 severity. Fatigue was the most frequent any grade and grade ≥3 TRAE. Safety results were consistent with other GITR monotherapy studies in which fatigue was among the most common TRAEs in most studies, and DLTs were reported in 0% to 8% of patients (45–48). INCAGN01876 monotherapy was associated with limited clinical efficacy in this population, among whom most (81%) had received ≥3 lines of prior systemic therapy. Only two confirmed partial responses were observed, in patients with appendiceal mucinous carcinoma and melanoma; however, the former partial response was maintained at the time of study termination (13.1 months). Because of a sponsor decision to focus the clinical development of INCAGN01876 on combination regimens with immune checkpoint inhibitors (ICI), enrollment in this study was terminated after 22 patients were enrolled in part 2 after Simon stage 1.

INCAGN01876 PK was found to be dose proportional for doses of 1 mg/kg or higher, which corresponds to a dose range within which the incidence of ADA was low; steady-state serum concentrations were generally reached at cycle 6. There was a direct relationship between INCAGN01876 serum concentration and the percentage of GITR receptor occupancy, which was described by a sigmoidal maximal effect model. INCAGN01876 doses of ≥5 mg/kg were sufficient to maintain 90% receptor occupancy at all time points for all patients. Although the MTD and MNTD were not reached and an RP2D was not assigned, safety, PK, and PD data from this study were used in combination with safety data from phase I of the phase I/II study of INCAGN01876 with nivolumab and/or ipilimumab (NCT03126110) to select an RP2D of 300 mg every 2 weeks used in phase II of this combination treatment study.

INCAGN01876 treatment was associated with increased expression of a number of plasma proteins, including the chemokine CCL17 and the immune-related ligand proteins CD70 and CLEC4G, which regulate T-cell activity and function. CCL17 is a chemokine for effector T cells and Tregs expressing CCR4 (42); CD70 is the ligand for CD27 (lymphocyte costimulatory receptor), which is a potent activator of T cells (41); and CLEC4G is a ligand for lymphocyte activation gene 3 (49), which is a T-cell coinhibitory receptor (40) and a CD4^+^ and CD8^+^ T-cell activation marker (50). In addition to these chemokines, BMP4 was also significantly upregulated upon INCAGN01876 treatment; of note, a previous study has indicated an association between BMP4 and CD4^+^ T-cell activation and IFN-γ suppression (51). The 5 mg/kg dose group demonstrated the most robust upregulation of CCL17; however, the 3 and 10 mg/kg dose groups demonstrated greater increases in CD70 and CLEC4G ligand protein expression. Thus, upregulation of these proteins in plasma provides evidence for immune-related INCAGN01876 activity; future studies of the mechanism(s) underlying these effects are warranted to better understand if upregulation of these proteins can serve as PD biomarkers of treatment response.

INCAGN01876 treatment resulted in a significant, although transient, decrease in levels of Tregs at C1D7 in the 5 mg/kg cohort. A trend of a decrease in Tregs was also observed in the 300 mg flat dosing cohort although the magnitude of the decrease did not reach significance. This similarity would be expected given that exposure resulting from a 300 mg flat dose is close to that for a 5 mg/kg dose for an average body weight of 60 to 70 kg. Of note, similar decreases in T-cell responses to those seen at 5 mg/kg and with the 300 mg flat dose were not observed in the other dose cohorts. This variance may reflect an apparent heterogeneity in the results of peripheral blood flow cytometry analysis, which may have resulted from differential or biphasic dose-dependent effects of INCAGN01876 on Treg depletion and T-cell expansion. Further experiments are required to delineate these two mechanisms of action. Nevertheless, these findings indicate that the transient depletion of Tregs in peripheral blood occurs only within a narrow dose and time window. It is possible that GITR^+^ Treg depletion resulting from INCAGN01876 treatment may involve tumor-specific ADCC via the Fc domain of the anti-GITR mAb. Such a mechanism has been proposed for the anti-mouse GITR mAb, DTA-1, in which agonistic binding of DTA-1 to GITR on Tregs is coupled with functional Fc receptor activation for Treg depletion (31). Here, INCAGN01876 treatment was associated with T-cell activation, based on observed increases in HLA-DR on T cells, and a trend of decrease in GITR^+^ Treg, which coincided with a trend of increase in CD8^+^ Ki67^+^ T cells both in peripheral blood and tumor samples from a subset of patients. The observation that EM CD4^+^ T cells were significantly, although transiently, decreased at a higher INCAGN01876 dose of 10 mg/kg every 2 weeks at C1D7, whereas central memory CD4^+^ T cells were only moderately increased, is notable and warrants future investigation. Of note, because EM T cells can migrate into inflammatory tissues (52), it is possible that some EM T cells may have infiltrated from the periphery into the tumor microenvironment upon INCAGN01876 treatment. It is also notable that when examined in a subset of patients, tumor infiltration of proliferating and activated T cells increased following INCAGN01876 treatment, with CD8^+^/Treg ratios increasing in the majority of these patients. Further studies are needed to corroborate this finding and clarify why these effects are not consistently observed in all patients.

Other GITR agonists in phase I clinical development include TRX518 (aglycosyl Fc defective IgG1 mAb, NCT02628574), GWN323 (IgG1 mAb, NCT02740270), MK-4166 (IgG1 mAb, NCT02132754), and MEDI1873 (trimeric GITRL with IgG1 domain, NCT0258165). In a phase I study of TRX518 for the treatment of advanced solid tumors (46), 1 out of 31 evaluable patients, who had previously treated hepatocellular carcinoma, achieved a partial response and remained progression-free for 685 days; 21 patients had stable disease, of whom two had stable disease lasting >6 months. Overall reductions in peripheral Tregs, effector Tregs, and GITR^+^ Tregs were observed during TRX518 treatment. A phase I study of single-agent GWN323 in patients with advanced or metastatic solid tumors reported stable disease in 7 of 39 evaluable patients although the study did not demonstrate significant T-cell activation (48). Another phase I study assessed MK-4166 for the treatment of advanced solid tumors (*N* = 113) and reported no objective responses in patients receiving monotherapy and five complete and three partial responses among 13 immunotherapy-naïve patients with melanoma receiving MK-4166 plus pembrolizumab (47). A phase I trial of MEDI1873 for the treatment of advanced solid tumors (*N* = 40) reported one unconfirmed partial response and stable disease in 17 patients, eight of whom had stable disease of duration ≥24 weeks (45). Treatment with MEDI1873 was associated with increased peripheral CD4^+^ effector T-cell proliferation, increased levels of cytokines that mediate effector T-cell activation, and reduced Treg activity.

The present results suggest that INCAGN01876 monotherapy induced immune modulation, which enhanced antitumor immunity. However, the observed effects on T-cell expansion and function may be insufficient to induce full immune activation, which might, in part, explain the observed limited efficacy associated with INCAGN01876 monotherapy. Limited observed efficacy may also be a consequence of nearly 50% of all enrolled patients having received and relapsed on prior anti–PD-1 mAb or anti–PD-L1 mAb therapy. The reduced INCAGN01876 exposure in some ADA-positive versus ADA-negative patients may also have led to limited antitumor activity. In addition, patients in this phase I study were not selected based on the presence of GITR pathway activation biomarkers, which may include GITR upregulation, activation markers (e.g., Ki76, HLA-DR), and/or markers of altered Treg function (e.g., cytokines; ref. 10). Future studies designed to enroll patients on the basis of such biomarkers are warranted to identify those who may derive treatment benefits from agonist GITR mAbs, including INCAGN01876.

The interplay between the distinct cellular mechanisms of GITR agonists, including INCAGN01876, and those of ICIs suggests that coadministration of INCAGN01876 with these agents may result in synergistic or additive immunomodulatory effects. Supporting this, combining GITR agonists with ICIs, including nivolumab and pembrolizumab, has been shown to provide improved efficacy in patients with advanced solid tumors (46, 47). Further evidence is provided by a recent study that demonstrated that a bispecific anti–PD-1 mAb-multimeric GITRL conjugate induced PD-1–dependent GITR clustering, enhanced GITR^+^ PD-1^+^ T-cell activation, and inhibited tumor growth in a mouse model (53). In this regard, GITR agonists may enhance ICI efficacy by reducing ICI resistance. Data from preclinical studies showed benefits of GITR agonist–ICI combinations in glioblastoma—a tumor found to be unresponsive to ICIs in phase III randomized controlled trials to date. In a preclinical study, GITR agonists promoted the conversion of Treg to effector T cells, inducing potent antitumor effects and reducing resistance to PD-1 treatment providing further support for the investigation of GITR agonist–PD-(L)1 combinations (54).

The representativeness of patients enrolled in this first-in-human study is presented in Supplementary Table S17. Study limitations include the smaller-than-planned number of patients enrolled in part 2, which limited the ability to fully evaluate the efficacy and safety of INCAGN01876 in the part 2 cohorts that enrolled patients with adenocarcinoma of the endometrium, melanoma, NSCLC, and RCC. These tumors may well be particularly sensitive to GITR blockade due to the high frequency of GITR-expressing tumor-infiltrating Tregs, particularly in melanoma, NSCLC, and RCC.

In conclusion, INCAGN01876 treatment was generally well tolerated at the studied doses in patients with advanced or metastatic solid tumors, with fatigue being the most frequent TEAE. Efficacy, involving effects on T-cell expansion and function, may be improved in select tumor types by combining INCAGN01876 with other immunotherapeutic and/or targeted agents.

## Supplementary Material

Supplementary data1 Supplementary materials
