# Supplementary material for: First-in-Human Phase I/II Study of INCAGN01876, a Glucocorticoid-Induced Tumor Necrosis Factor Receptor Agonist, in Patients with Advanced or Metastatic Solid Tumors
Source: Clin Cancer Res. 2025 Jul 25;31(19):4089–100. doi: 10.1158/1078-0432.CCR-24-4141 (PMC12485371; doi:10.1158/1078-0432.CCR-24-4141)

SUPPLEMENTAL MATERIALS

First-in-Human Phase 1/2 Study of INCAGN01876, a Glucocorticoid-Induced Tumor Necrosis Factor Receptor Agonist, in Patients With Advanced or Metastatic Solid Tumors

Omid Hamid,[1] Dana B. Cardin,[2] F. Stephen Hodi,[3] Patricia LoRusso,[4] Taha Merghoub,[5] Roberta Zappasodi,[5] Rachana Maniyar,[5] John E. Janik,[6] Maikel van der Velden,[7] Feng Zhou,[6] Zhiwan Dong,[6] Xuejun Chen,[6] James J. Harding,[8,9]

The Angeles Clinic and Research Institute, A Cedars Sinai Affiliate, Los Angeles, California, USA; Division of Hematology and Oncology, Department of Internal Medicine, Vanderbilt University Medical Center, Nashville, TN, USA; Dana-Farber Cancer Institute, Boston, MA, USA; Yale Cancer Center, New Haven, CT, USA; Meyer Cancer Center, Weill Cornell Medicine, Weill Cornell Medical College, New York, NY, USA; Incyte Corporation, Wilmington, DE, USA; Incyte Biosciences International Sàrl, Morges, Switzerland; Department of Medicine, Memorial Sloan Kettering Cancer Center, New York, NY, USA; Weill Medical College at Cornell University, New York, NY, USA.

Contents

Supplemental Methods **3**
Rationale for part 2 patient population 3
Representative FC plots for tumors with intratumoral regulatory T-cells which consistently expressed elevated levels of GITR 3
Rationale for starting dose 3
Dose escalation 4
Maximum number of tolerated doses 5
Pharmacokinetic and antidrug antibody assessments 5
Receptor occupancy analysis 5
Circulating immune cell analysis 6
Simon two-stage design 6
Supplemental Results **7**
Safety 7
Supplemental Tables **8**
Supplemental Table 1. Definition of dose-limiting toxicity. 8
Supplemental Table 2. Multicolor flow cytometry panel used for analysis of markers of memory/naïve T-cell subsets, Tregs, and T-cell activation/exhaustion. 9
Supplemental Table 3. FACS panel used for circulating immune cell assays at MSK. 10
Supplemental Table 4. Multiplex IHC panel used for analysis of tumor infiltrating lymphocytes. 11
Supplemental Table 5. 7-plex Vectra immunofluorescence panel used for analysis of tumor infiltrating lymphocytes at MSK. 12
Supplemental Table 6. Baseline demographics and characteristics of the MSK cohort of patients analyzed longitudinally for circulating immune cells in PBMCs by FACS (*n* = 8), and tumor infiltration of T cells by IHC (*n* = 7). 13
Supplemental Table 7. Baseline demographics and characteristics of patients with available paired biopsy samples for analysis of tumor infiltrating lymphocytes using a 9-color multiplex IHC panel. 14
Supplemental Table 8. Treatment-related TEAEs. 15
Supplemental Table 9. Incidence and management of skin-related TEAEs in patients requiring concomitant medicines. 20
Supplemental Table 10. Grade ≥3 TRAEs and exposure. 22
Supplemental Table 11. Fatal TEAEs. 23
Supplemental Table 12. TEAEs leading to treatment discontinuation. 24
Supplemental Table 13. TEAEs leading to treatment interruption. 25

Supplemental Table 14. Descriptive statistics of the pharmacokinetic parameters following the first dose (cycle 1) or at steady state (cycle 6). 26
Supplemental Table 15. Incidence of antidrug antibodies to INCAGN01876. 28
Supplemental Table 16. Best overall responses based on RECIST v1.1 and mRECIST. 29
Supplemental Table 17. Representativeness of study participants. 30
Supplemental Figures 32
Supplemental Fig. 1. Study design. 32
Supplemental Fig. 2. INCAGN01876 individual predose trough concentrations versus time since first dose by dosing regimen. 33
Supplemental Fig. 3. Select INCAGN01876 individual concentration–time profiles at cycle 1 and cycle 6 depicting patients with ADA-positive and ADA-negative samples. 34
Supplemental Fig. 4. (A) Cytokine upregulation on INCAGN01876 treatment. 35
(B) Relationship between INCAGN01876 dose and cytokine upregulation. 35
Supplemental Fig. 5. Changes in frequency of (A) total and (B, D) GITR-expressing Tregs and (C, E) peripheral Ki67$^+$ CD8$^+$ T cells in the MSK cohort following INCAGN01876 treatment. 36
Supplemental Fig. 6. GITR$^+$ FoxP3$^+$ flow cytometry gating strategy data for GITR+FoxP3+ populations. Baseline examples are shown for the patient receiving INCAGN01876 5mg/kg Q2W indicated in Supplemental Figure 5. 37
Supplemental Fig. 7. Representative flow plots for GITR$^+$ FoxP3$^+$ cells showing reduction in GITR$^+$ FoxP3$^+$ cells over time from the patient receiving INCAGN01876 5 mg/kg Q2W indicated in Supplemental Figure 5. 38
Supplemental Fig. 8. Tumor infiltration of T cells in paired biopsies. 39
Supplemental Fig. 9. Tumor infiltration of T cells in paired biopsies from MSK analysis. 40
Supplemental Fig. 10. Representative T cell infiltration biopsy immunofluorescence images from two patients, each showing two fields of view. 41

Supplemental Methods

Rationale for part 2 patient population

Preclinical evaluations of INCAGN01876 support three potential antitumor mechanisms of action: 1) costimulatory agonistic engagement of GITR enhancing T effector cells, 2) depletion of Tregs via the induction of lineage instability, and 3) co-engagement of activating FcγRs to selectively deplete immune suppressive Tregs located within the tumor. Together these mechanisms would suggest that an agonistic GITR mAb, such as INCAGN01876 would have a focused effect in the human immune response leading to the proliferation and augmentation of an already active immune response. To test this hypothesis four tumor histologies (adenocarcinoma of the endometrium, melanoma, non-small cell lung cancer [NSCLC], and renal cell carcinoma [RCC]), that may be most susceptible to an agonistic mAb to GITR were selected for evaluation in part 2 of this study. Melanoma and NSCLC were selected and evaluated because they are both immunogenic histologies that have been proven to respond to checkpoint inhibitors (CTLA-4 and PD1)(37). Additionally, preclinical data evaluating a variety of tumor histologies demonstrated that endometrial cancer, NSCLC, and RCC, consistently had intratumoral regulatory T cells with elevated GITR expression. Here, dissociated (frozen) primary tumor samples were analyzed by flow cytometry (FC). After gating on either $CD4^+$ $CD25^-$ $FoxP3^-$ (Teff cells) and $CD4^+$ $CD25^+$ $FoxP3^+$ (Treg), GITR expression was analyzed (see representative FC plots below). Based on these data, endometrial cancer, melanoma, NSCLC, and RCC were selected to be evaluated in part 2 of this study.

Representative FC plots for tumors with intratumoral regulatory T-cells which consistently expressed elevated levels of GITR

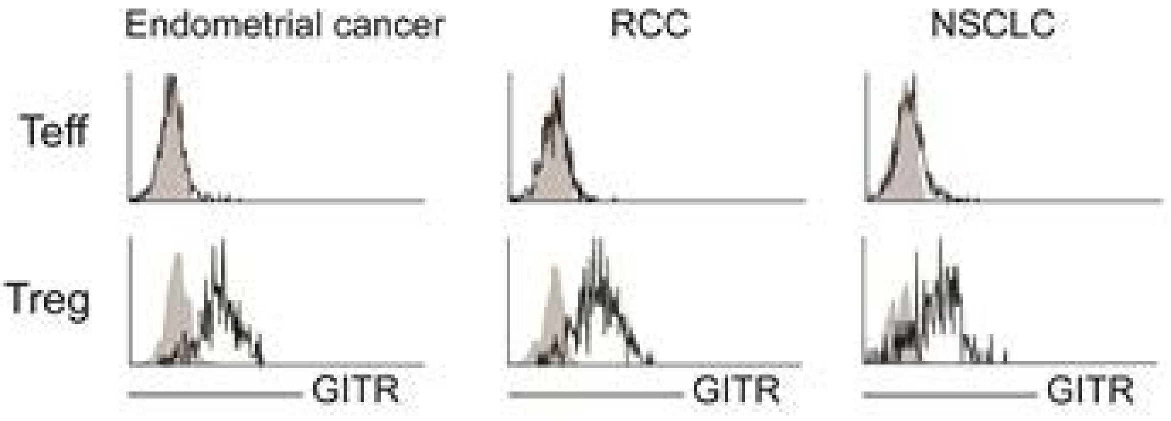

Rationale for starting dose

The INCAGN01876 safe starting dose (SSD) was based on all relevant in vitro and in vivo data and using a weight of evidence approach. The primary data sets used in deriving the SSD included in vitro assessment of GITR agonist activity (cytokine release) in human T cells, in vivo

assessment of toxicity in the 4-week study in African green monkeys (AGMs), and in vivo exposure data derived from the study in AGMs (Incyte Corporation, data on file). The SSD is also supported by data that demonstrate: 1) peripheral blood T-cells do not express appreciable levels of GITR, 2) enhanced cytokine release only occurred in the presence of agents that sub-optimally stimulated the T-cell receptor, and 3) that the expression of GITR and the potency of INCAGN01876 in the cytokine assays was similar between human and AGMs. Together, these data support that GITR agonism would not be expected to result in broad activation of the immune system. The anti-GITR agonist activity of INCAGN01876 was evaluated in in vitro studies investigating the ability of INCAGN01876 to activate the GITR signaling pathway in T cells. The in vitro assays that assessed the impact of INCAGN01876 agonist signaling to elicit enhanced cytokine release in primary human $CD4^+$ and $CD8^+$ T cells were considered most relevant to the assessment of potential safety signals in humans and to the determination of the SSD due to the potential of cytokines released from activated T cells to result in cytokine release syndrome. Assays that characterized potential antibody-dependent cell-mediated cytotoxicity and antibody-dependent cell-mediated phagocytosis through FcγR engagement were not factored into the determination of the SSD, as they were based on artificial/engineered in vitro systems and considered to not be representative of potential safety risks or in vivo responses. The relevant assays and associated EC_{50} values are presented in the table below. The SSD of 0.03 mg/kg is a dose that is considered an approximate minimum anticipated biological effect level (based on cytokine release in human T cells) and is 417-fold lower than the potency-adjusted no-observed-effect level determined for the AGM, and four-fold lower than the estimated EC_{50} for cytokine release. The proposed SSD attempts to minimize the exposure of patients to subtherapeutic dose levels of INCAGN01876 while balancing safety risk associated with the nonclinical profile of the agent. Fixed dosing was chosen for benefits over weight-based dosing including convenience of preparation and administration, reduced errors in preparation calculation, and minimization of drug waste.

Estimated EC_{50} values in human T cells

	**~EC_{50} (µg/mL)**
INCAGN01876-mediated NFκB activation in T cells (no TCR)	3.0
INCAGN01876-mediated NFκB activation in T cells (with TCR)	1.9
INCAGN01876-induced human CD8 T-cell IFNγ production (with TCR)	3.3
INCAGN01876-induced human CD4 T-cell IFNγ production (with TCR)	3.5
INCAGN01876-induced human TNFα (supernatant; with TCR)	2.0
Mean human T-cell cytokine release EC_{50} value (µg/mL)	2.7
- Mean human T-cell cytokine release EC_{50} value converted to dose (mg/kg)[a]	0.12

TCR, T-cell receptor stimulation. Based on TK ($C_{average}$) from a 4-week study in AGMs: AUC_{0-143h} = 3790 µg·h/mL at 1 mg/kg; average concentration ($C_{average}$) = 3790/(7 × 24 hours) = 22.6 µg/mL.

Dose escalation

Per the 3+3 design a minimum of three evaluable patients were enrolled in each cohort beginning with cohort 1 (0.03 mg/kg; starting dose), with a waiting period of 48 h between dose administration of the first and second patient of each dosing cohort. The first three evaluable patients within a cohort were observed for a dose limiting toxicity (DLT) observation period of

28 days, before the next cohort began enrollment. The dose was escalated if none of the first three evaluable patients enrolled had a DLT. If one of the first three evaluable patients enrolled had a DLT, then the cohort was expanded to include three additional evaluable patients, and if no DLT occurred in the additional three patients, then the dose was escalated. If a DLT occurred in one-third or more of the expanded cohort, then the MTD was deemed to be exceeded and the previous dose level considered the MTD. If a DLT occurred at the 20.0 mg/kg dose, then a dose level of 15.0 mg/kg could be explored pending safety review. If only three patients were treated at the MTD or pharmacologically active dose, then a minimum of three additional evaluable patients would be enrolled at this dose before it was administered in part 2 of the study. If cohort 1 (0.03 mg/kg; starting dose) exceeded the MTD, the sponsor and investigators would consider dosing INCAGN01876 at 0.01 mg/kg (cohort -1), and/or investigate 0.03 mg/kg at alternate dose schedules, based on available safety, PK, PD, and biomarker data. If an alternate schedule were tested and determined to be safe, re-escalation of INCAGN01876 would proceed. Additional patients would be enrolled in a cohort to achieve the minimum of three evaluable patients. Patients who drop out for reasons other than a DLT (e.g., events clearly associated with the underlying disease, disease progression, concomitant medication, or comorbidity), during the 28-day DLT observation period were considered non-evaluable and replaced. Dose modifications were not allowed during the DLT observation period without discussion with the medical monitor. Intrapatient dose escalation was not permitted; however, once the recommended part 2 dose (RP2D) and schedule were determined, ongoing patients in part 1 could escalate to the RP2D with approval of the medical monitor.

Maximum number of tolerated doses

Maximum number of tolerated doses would be met if during the treatment period > 33% of patients (a minimum of six) experience a ≥ grade 3 toxicity related to study drug after completing ≥ four cycles. Dose administration will be stopped, and the maximum number of tolerated doses will be determined in conjunction with the investigators and sponsor based on all available safety data.

Pharmacokinetic and antidrug antibody assessments

For PK analysis, a predose blood sample was drawn within 24 hours before drug infusion. During cycle 1 and cycle 6, samples were collected preinfusion on day 1, immediately postinfusion (±10 minutes), and then postinfusion at 4 hours during day 1, at 24 hours during day 2, and an unspecified time during day 7. Preinfusion samples were also collected during day 1 of cycle 2, 3, 4, and 7. Serum samples for measurement of antidrug antibodies (ADAs) were collected preinfusion during day 1 of cycle 1, 3 and 6, and at 30 days post-treatment.

Receptor occupancy analysis

Briefly, Jurkat cells expressing GITR were incubated for 30 min with plasma collected prior to treatment or at various time points following infusion. At the end of the 30 min incubation period, saturating concentration of fluorescently labeled INCAGN01876 was added and incubated for 15 min. Cells were subsequently washed and the ability of the fluorescently labeled INCAGN01876 to bind to cells was then analyzed by flow cytometry. The cycle 1 day 1 predose

samples were used as baseline and maximum occupancy was determined using the cycle 1 day 1 predose sample spiked with saturating concentration of unlabeled INCAGN01876.

Circulating immune cell analysis

Multicolor flow cytometry was used to evaluate the cellular biomarkers in peripheral blood. Frequencies of immune cells were monitored at screening and at various time points on treatment. Blood was collected in Cyto-Chex® collection tubes and shipped overnight to Caprion bioscience for immediate assessment using two flow panels evaluating T-cell subsets (memory / naïve and Treg) and T-cell function (activation/exhaustion). The two flow cytometry panels are described in **Supplemental Tables 2** and **3**, respectively. Samples from patients receiving doses ≥3 mg/kg were analyzed. Due to limited samples from the 20 mg/kg cohort, statistics were calculated only with data from 3 mg/kg, 5 mg/kg, 10 mg/kg, 300 mg and 400 mg cohorts.

Simon two-stage design

Nine patients were to be initially enrolled per cohort (Stage 1) to determine whether the target response rate of 25% is likely. If no responses are observed in a cohort, then a true response rate ≥25% would be concluded unlikely and enrollment in that cohort terminated at Stage 2. If there are ≥1 responses in a cohort, 8 additional evaluable patients would be enrolled at Stage 2. Probabilities of response were calculated based on a one-sided Type I error of 0.05 and power of 80% for each tumor cohort.

Supplemental Results

Safety

Five patients had TEAEs leading to treatment discontinuation, one was considered treatment related. Four of these five patients had experienced serious TEAEs. Of these, only one patient had been previously treated with an ICI, having received both durvalumab and tremelimumab, both treatments had been discontinued due to progressive disease **(Supplemental Table 12)**. Two patients experienced DLTs, both occurring after the second INCAGN01876 infusion. One patient in the 3 mg/kg Q2W cohort, experienced a grade 4 TEAE of hypoxia that was not considered treatment-related. This 42-year old White male with metastatic sarcoma who had undergone prior surgery, radiation, and systemic therapy, had a number of ongoing medical illnesses/symptoms, including wheezing. The patient was given concomitant medication; however, symptoms had not resolved by the end of the study. A second patient in the 5 mg/kg Q2W cohort, was diagnosed with a grade 4 AE of dyspnea due to pleuritis, which was deemed to be treatment-related. This 32-year old White female had metastatic ovarian cancer, had undergone prior surgery, radiation, and systemic therapy, and had a number of ongoing medical illnesses, including a left pleural effusion. Concomitant medication was given and the patient recovered after 14 days.

Supplemental Tables

Supplemental Table 1. Definition of dose-limiting toxicity.

Nonhematologic toxicity
• Any ≥grade 3 nonhematologic toxicity EXCEPT the following:<br>o Transient (≤72 hours) abnormal laboratory values without associated clinically significant signs or symptoms.<br>o Nausea, vomiting, and diarrhea adequately controlled with supportive care within 48 hours.<br>o Changes in cholesterol and triglycerides.<br>o An event clearly associated with the underlying disease, disease progression, a concomitant medication, or comorbidity.<br>o Asymptomatic changes in lipid profiles.<br>o Asymptomatic changes in amylase and lipase.<br>o Single or nonfasting elevations in blood glucose (i.e., blood glucose excursions were considered toxicities if fasting blood glucose is elevated on two separate occasions).
Hematologic toxicity
• Grade 3 thrombocytopenia with clinically significant bleeding (i.e., requires hospitalization, transfusion of blood products, or other urgent medical intervention).<br>• Grade 4 thrombocytopenia.<br>• ≥Grade 3 febrile neutropenia (absolute neutrophil count <1 × 10^9/L and fever >101°F/38.3°C).<br>• Grade 4 neutropenia that does not recover to ≤grade 2 in ≤3 days after interrupting study drug.<br>• Grade 4 anemia not explained by underlying disease or some other concomitant disorder.
Immune-related toxicity[a]
• ≥Grade 2 ocular irAEs were considered a DLT.<br>• Grade 3 irAEs that do not improve to baseline or at least grade 1 in <5 days with appropriate care or with corticosteroid therapy were considered a DLT.<br>• Grade 4 irAEs were considered a DLT regardless of duration.
General
• Inability to receive the planned number of doses within the 28-day DLT period due to toxicity, regardless of grade, was considered a DLT.
MTD
• In part 1 of the study, the MTD was defined as one dose level below that at which ≥one-third of patients in a particular cohort have DLTs.<br>• In part 2 of the study, toxicities continued to be monitored. If the cumulative incidence of DLTs occurs in >33% of patients after six patients have been observed for at least 28 days, then further enrollment could be interrupted, and the investigators and sponsor met and reassessed the MTD. All AEs, regardless of the time of occurrence on study, could be considered in determining the appropriate dose, schedule, and MNTD.
MNTD
• Throughout the treatment period, if >33% of patients (minimum of six patients) experienced a ≥grade 3 toxicity related to study drug after completing ≥4 cycles, then dose administration was stopped, and the MNTD was determined in conjunction with the investigators and sponsor based on all available safety data.

[a] Immune-related AEs are a diagnosis of exclusion, after alternative etiologies have been ruled out.
Abbreviations: AE, adverse event; DLT, dose-limiting toxicity; irAEs, immune-related AEs; MNTD, maximum number of tolerated doses; MTD, maximum tolerated dose.

Supplemental Table 2. Multicolor flow cytometry panel used for analysis of markers of memory/naïve T-cell subsets, Tregs, and T-cell activation/exhaustion.

**Antibody**[a]	**RRID**
**Markers for memory/naïve T-cell subsets and Tregs**	
Live/dead	(ThermoFisher Scientific Cat# L34957)
CD3	(BD BioSciences Cat#564001)
CD4	(BD Biosciences Cat# 563028, RRID:AB_2737961)
CD8 – BV 650	(BD Biosciences Cat# 563821, RRID:AB_2744462)
CD25	(BD Biosciences Cat# 341009, RRID:AB_400203)
CD28 – PerCP – Cy5.5	(BioLegend Cat# 302922, RRID:AB_2073718)
CD45RA – APC – Cy7	(BioLegend Cat# 304128, RRID:AB_10708880)
CD45RO	(BD BioSciences Cat# 562791)
CD95	(BD Biosciences Cat# 564710, RRID:AB_2738907)
CD127 – BV 421	(BioLegend Cat# 351310, RRID:AB_10960140)
CCR7 – PE – Cy7	(BioLegend Cat# 353226, RRID:AB_11126145)
FoxP3 - APC	(Thermo Fisher Scientific Cat# 17-4776-42, RRID:AB_1603280)
Ki67 - FITC	(BD Biosciences Cat# 556026, RRID:AB_396302)
**Markers for T-cell activation/exhaustion markers**	
Live/dead	(ThermoFisher Scientific Cat# L34957)
CD3	(BD BioSciences Cat#564001)
CD4	(BD Biosciences Cat# 563028, RRID:AB_2737961)
CD8 – BV 650	(BD Biosciences Cat# 563821, RRID:AB_2744462)
CD38	(BD Biosciences Cat# 551400, RRID:AB_394184)
CD244	(BD Biosciences Cat# 564881, RRID:AB_2738999)
CD137 - APC	(BD Biosciences Cat# 550890, RRID:AB_398477)
HLA-DR – BV 785	(BioLegend Cat# 307642, RRID:AB_2563461)
ICOS	(BD Biosciences Cat# 564549, RRID:AB_2738840)
PD-1 – BV 605	(BioLegend Cat# 329924, RRID:AB_2563212)
TIM-3 - PE	(BioLegend Cat# 345006, RRID:AB_2116576)
LAG-3 – PE – Cy7	(Thermo Fisher Scientific Cat# 25-2239-42, RRID:AB_2573430)
CTLA-4	(BD Biosciences Cat# 562743, RRID:AB_2737762)

[a]Conjugate fluorophore is included where available.

RRID, Research Resource Identification (see https://rrid.site/).

Supplemental Table 3. FACS panel used for circulating immune cell assays at MSK.

Marker[a]	RRID
CD45 RA – V500	(BD Biosciences Cat# 561640, RRID:AB_10896479)
Foxp3 – eFluor 450	(Thermo Fisher Scientific Cat# 48-4776-42, RRID:AB_1834364)
CD8a – BV 570	(BioLegend Cat# 301038, RRID:AB_2563213)
CD4 – Qdot 605	(Thermo Fisher Scientific Cat# Q10008 RRID:AB_11180611)
CD134/OX40 – BV 650	(BD Biosciences Cat# 563658, RRID:AB_2738353)
CD3 - FITC	(BioLegend Cat#300440 RRID:AB_2562046)
GITR – PerCP – eFluor 710	(Thermo Fisher Scientific Cat# 46-5875-42, RRID:AB_2573785)
CD127 – PE – CF594	(BD Biosciences Cat# 562397, RRID:AB_11154212)
PD-1 - PE	(Thermo Fisher Scientific Cat# 12-2799-42, RRID:AB_11042478)
CD56 – PE – Cy5	(Thermo Fisher Scientific Cat# 15-0567-42, RRID:AB_2573062)
CD25 – PE – Cy7	(BD Biosciences Cat# 560920, RRID:AB_10562555)
CCR7 – AlexaFluor 647	(BD Biosciences Cat# 560816, RRID:AB_2033948)
Ki67 – AlexaFluor 700	(BD Biosciences Cat# 561277, RRID:AB_10611571)
Live/dead	(ThermoFisher Scientific Cat# L10119)
CD19 – APC Cy7	(BioLegend Cat# 302218 RRID:AB_314248)

[a]Conjugate fluorophore is included where available.

FACS, fluorescence-activated cell sorting; MSK, Memorial Sloan Kettering; RRID, Research Resource Identification (see https://rrid.site/).

Supplemental Table 4. Multiplex IHC panel used for analysis of tumor infiltrating lymphocytes.

**Round**	**Cyanine3 labeled markers**
1	PanCK
2	S100
3	CD8
4	CD4
5	CD3
**Round**	**Cyanine5 labeled markers**
1	CD68
2	HLA_DR
3	FOXP3
4	Ki67

Supplemental Table 5. 7-plex Vectra immunofluorescence panel used for analysis of tumor infiltrating lymphocytes at MSK.

Marker	RRID
Ki67	(Biocare Medical Cat# CRM 325, RRID:AB_2721189)
GITR	(Cell Signaling Technology Cat# 68014, RRID:AB_2799740)
CD8	(Cell Signaling Technology Cat# 70306, RRID:AB_2799781)
Foxp3	(Biocare Medical Cat # API3164AA, clone 236A/E7)
CD3	(Biocare Medical Cat # ACI3170A, clone BC33)
PanCK	(Agilent Cat# M3515, RRID:AB_2132885)
DAPI	NA

NA, not available; RRID, Research Resource Identification (see https://rrid.site/).

Supplemental Table 6. Baseline demographics and characteristics of the MSK cohort of patients analyzed longitudinally for circulating immune cells in PBMCs by FACS (*n* = 8), and tumor infiltration of T cells by IHC (*n* = 7).

Pt#	Dose (mg/kg Q2W)	Tumor type	Age	Sex	Race	ECOG PS	Prior therapy				
							Platinum-therapy	Radiotherapy	Surgery	Immunotherapy	Anti–PD-1 or PD-L1 therapy
1[a]	10	Pancreatic	76	M	White	1	Yes	No	No	Yes	No
2[ab]	5	Colon	62	M	White	0	No	No	Yes	Yes	Yes
3[†ab]	5	Breast	50	F	Missing	1	No	Yes	Yes	Yes	No
4[a]	5	Colorectal	59	F	White	1	Yes	No	Yes	Yes	Yes
5[b]	3	CCA	67	F	White	0	Yes	No	No	No	No
6[a]	5	Breast	53	F	White	1	No	Yes	Yes	Yes	No
7[ab]	10	Colorectal	70	M	White	0	Yes	No	Yes	Yes	No
8[ab]	3	Pancreatic	58	F	White	0	No	Yes	Yes	Yes	No
9[ab]	3	HCC	82	F	White	0	No	No	Yes	Yes	No
10[b]	5	Anal	59	F	Black	0	Yes	Yes	Yes	Yes	Yes

[a] Patients had PBMC samples for FACS analysis of circulating immune cells**;** [b]Patients had paired biopsy for IHC analysis of tumor infiltrating T cells.
Abbreviations: CCA, cholangiocarcinoma; ECOG PS, Eastern Cooperative Oncology Group performance status; F, female; FACS, fluorescence-activated cell sorting; HCC, hepatocellular cancer; MSK, Memorial Sloan Kettering Cancer Center; M, male; PD-1, programmed cell death protein 1; PD-L1, programmed death ligand 1; PBMC, peripheral blood mononuclear cell; Q2W, every 2 weeks.

Supplemental Table 7. Baseline demographics and characteristics of patients with available paired biopsy samples for analysis of tumor infiltrating lymphocytes using a 9-color multiplex IHC panel.

							Prior therapy				
Pt#	Dose (mg/kg Q2W)	Tumor type	Age	Sex	Race	ECOG PS	Platinum-therapy	Radiotherapy	Surgery	Immunotherapy	Anti–PD-1 or PD-L1 therapy
1	3	H&NC	68	M	White	1	Yes	Yes	No	Yes	Yes
2	5	CCA	27	F	Missing	0	Yes	[b]	No	-[b]	-[b]
3	300	Melanoma	35	M	White	0	Yes	-[b]	Yes	Yes	Yes
4	400[a]	Melanoma	76	F	White	0	Yes	-[b]	No	Yes	Yes
5	300	Melanoma	78	F	White	0	Yes	Yes	Yes	Yes	Yes
6	5	UTC/BC	62	M	White	0	Yes	Yes	Yes	-[b]	-[b]
7	3	NSCLC	67	M	Black	1	Yes	Yes	Yes	Yes	Yes
8	10	NSCLC	57	F	White	1	Yes	Yes	Yes	Yes	Yes
9[c]	300	RCC	72	M	White	1	Yes	Yes	Yes	Yes	Yes
10	300	NSCLC	74	M	White	1	Yes	Yes	No	Yes	Yes

[a]400 mg dose is Q4W. [b]Missing treatment data. [c]Patient excluded from summary due to negative tumor marker (CK) staining.

Abbreviations: BC, bladder cancer; CCA, cholangiocarcinoma; CK, cytokeratin; ECOG PS, Eastern Cooperative Oncology Group performance status; F, female; H&NC, head and neck cancer; IHC, immunohistochemistry; M, male; NSCLC, non—small cell lung cancer; PD-1, programmed cell death protein-1; PD-L1, programmed death ligand-1; Q2W, every 2 weeks; Q4W, every 4 weeks; RCC, renal cell carcinoma; UTC, urothelial tract cancer.

Supplemental Table 8. Treatment-related TEAEs.

Adverse Event,[a] *n* (%)	Treatment group															
	0.03 to 1 mg/kg Q2W (*n* = 15)		3 mg/kg Q2W (*n* = 15)		5 mg/kg Q2W (*n* = 18)		10 mg/kg Q2W (*n* = 16)		20 mg/kg Q2W (*n* = 4)		400 mg Q4W (*n* = 10)		300 mg Q2W (*n* = 22)		Total (*N* = 100)	
	Any Gr	Gr ≥3	Any Gr	Gr ≥3	Any Gr	Gr ≥3	Any Gr	Gr ≥3	Any Gr	Gr ≥3	Any Gr	Gr ≥3	Any Gr	Gr ≥3	Any Gr	Gr ≥3
Fatigue	1 (6.7)	0	4 (26.7)	0	2 (11.1)	0	1 (6.3)	0	2 (50.0)	1 (25.0)	2 (20.0)	0	5 (22.7)	2 (9.1)	17 (17.0)	3 (3.0)
Pruritus	1 (6.7)	0	4 (26.7)	0	3 (16.7)	0	3 (18.8)	0	0	0	1 (10.0)	1 (10.0)	2 (9.1)	0	14 (14.0)	1 (1.0)
Generalized pruritus	1 (6.7)	0	3 (20)	0	1 (5.6)	0	0	0	0	0	2 (20.0)	0	1 (4.5)	0	8 (8.0)	0
Generalized rash	1 (6.7)	0	2 (13.3)	0	2 (11.1)	0	1 (6.3)	0	0	0	1 (10.0)	0	1 (4.5)	0	8 (8.0)	0
Decreased appetite	0	0	1 (6.7)	0	0	0	1 (6.3)	0	2 (50.0)	0	1 (10.0)	0	2 (9.1)	0	7 (7.0)	0
Abdominal pain	1 (6.7)	0	2 (13.3)	0	0	0	2 (12.5)	0	0	0	1 (10.0)	0	0	0	6 (6.0)	0
Dyspnea	1 (6.7)	0	1 (6.7)	1 (6.7)	2 (11.1)	1 (5.6)	0	0	0	0	1 (10.0)	0	1 (4.5)	0	6 (6.0)	2 (2.0)
Diarrhea	1 (6.7)	0	4 (26.7)	1 (6.7)	0	0	0	0	0	0	0	0	0	0	5 (5.0)	1 (1.0)
Anemia	0	0	2 (13.3)	1 (6.7)	0	0	1 (6.3)	0	0	0	0	0	1 (4.5)	1 (4.5)	4 (4.0)	2 (2.0)
Vomiting	0	0	2 (13.3)	0	0	0	0	0	1 (25.0)	0	0	0	1 (4.5)	0	4 (4.0)	0
Nausea	0	0	0	0	2 (11.1)	0	1 (6.3)	0	0	0	0	0	1 (4.5)	0	4 (4.0)	0
Rash	0	0	1 (6.7)	0	1 (5.6)	0	2 (12.5)	0	0	0	0	0	0	0	4 (4.0)	0
Maculo-papular rash	0	0	2 (13.3)	0	0	0	0	0	0	0	1 (10.0)	0	1 (4.5)	0	4 (4.0)	
Gastrointestinal reflux disease	0	0	1 (6.7)	0	0	0	1 (6.3)	0	0	0	0	0	1 (4.5)	0	3 (3.0)	0

Pyrexia	0	0	1 (6.7)	0	1 (5.6)	0	0	0	0	0	0	0	1 (4.5)	0	3 (3.0)	0
Alanine aminotransferase increased	0	0	1 (6.7)	1 (6.7)	1 (5.6)	0	0	0	0	0	0	0	1 (4.5)	0	3 (3.0)	1 (1.0)
Flatulence	1 (6.7)	0	0	0	0	0	1 (6.3)	0	0	0	0	0	0	0	2 (2.0)	0
Hyperthyroidism	0	0	0	0	0	0	1 (6.3)	0	0	0	0	0	1 (4.5)	0	2 (2.0)	0
Peripheral edema	0	0	0	0	2 (11.1)	0	0	0	0	0	0	0	0	0	2 (2.0)	0
Chills	0	0	1 (6.7)	0	0	0	0	0	0	0	0	0	1 (4.5)	0	2 (2.0)	0
Hypersensitivity	0	0	0	0	2 (11.1)	0	0	0	0	0	0	0	0	0	2 (2.0)	0
Aspartate aminotransferase increased	0	0	1 (6.7)	1 (6.7)	0	0	0	0	0	0	0	0	1 (4.5)	0	2 (2.0)	1 (1.0)
Cough	0	0	1 (6.7)	1 (6.7)	1 (5.6)	0	0	0	0	0					2 (2.0)	1 (1.0)
Arthralgia	0	0	1 (6.7)	0	1 (5.6)	0	0	0	0	0	0	0	0	0	2 (2.0)	0
Myalgia	0	0	1 (6.7)	0	1 (5.6)	0	0	0	0	0	0	0	0	0	2 (2.0)	0
Headache	1 (6.7)	0	1 (6.7)	0	0	0	0	0	0	0	0	0	0	0	2 (2.0)	0
Iron deficiency anemia	1 (6.7)	0	0	0	0	0	0	0	0	0	0	0	0	0	1 (1.0)	0
Lymph node pain	1 (6.7)	0	0	0	0	0	0	0	0	0	0	0	0	0	1 (1.0)	0
Thrombocytopenia	0	0	1 (6.7)	0	0	0	0	0	0	0	0	0	0	0	1 (1.0)	0
Cardiac tamponade	0	0	1 (6.7)	1 (6.7)	0	0	0	0	0	0	0	0	0	0	1 (1.0)	1 (1.0)
Constrictive pericarditis	0	0	1 (6.7)	1 (6.7)	0	0	0	0	0	0	0	0	0	0	1 (1.0)	1 (1.0)

Hypothyroidism	0	0	0	0	0	0	1 (6.3)	0	0	0	0	0	0	0	1 (1.0)	0
Photophobia	0	0	1 (6.7)	0	0	0	0	0	0	0	0	0	0	0	1 (1.0)	0
Dyspepsia	1 (6.7)	0	0	0	0	0	0	0	0	0	0	0	0	0	1 (1.0)	0
Localized edema	1 (6.7)	0	0	0	0	0	0	0	0	0	0	0	0	0	1 (1.0)	0
Generalized edema	1 (6.7)	0	0	0	0	0	0	0	0	0	0	0	0	0	1 (1.0)	0
Constipation	0	0	1 (6.7)	0	0	0	0	0	0	0	0	0	0	0	1 (1.0)	0
Dry mouth	0	0	0	0	0	0	1 (6.3)	0	0	0	0	0	0	0	1 (1.0)	0
Asthenia	0	0	1 (6.7)	0	0	0	0	0	0	0	0	0	0	0	1 (1.0)	0
Influenza-like illness	0	0	0	0	0	0	0	0	1 (25.0)	0	0	0	0	0	1 (1.0)	0
Malaise	0	0	1 (6.7)	0	0	0	0	0	0	0	0	0	0	0	1 (1.0)	0
Noncardiac chest pain	0	0	0	0	1 (5.6)	0	0	0	0	0	0	0	0	0	1 (1.0)	0
Conjunctivitis	0	0	0	0	1 (5.6)	0	0	0	0	0	0	0	0	0	1 (1.0)	0
Radiation pneumonitis	0	0	0	0	0	0	0	0	0	0	0	0	1 (4.5)	0	1 (1.0)	0
Amylase increased	0	0	1 (6.7)	0	0	0	0	0	0	0	0	0	0	0	1 (1.0)	0
Blood alkaline phosphatase increased	0	0	0	0	1 (5.6)	0	0	0	0	0	0	0	0	0	1 (1.0)	0
Blood creatine increased	0	0	0	0	1 (5.6)	0	0	0	0	0	0	0	0	0	1 (1.0)	0
Gamma-glutamyltransferase increased	0	0	1 (6.7)	0	0	0	0	0	0	0	0	0	0	0	1 (1.0)	0

Lipase increased	0	0	1 (6.7)	0	0	0	0	0	0	0	0	0	0	0	1 (1.0)	0
Transaminases increased	0	0	0	0	1 (5.6)	0	0	0	0	0	0	0	0	0	1 (1.0)	0
Weight decreased	0	0	0	0	0	0	0	0	0	0	0	0	1 (4.5)	0	1 (1.0)	0
Dehydration	0	0	0	0	0	0	0	0	1 (25.0)	0	0	0	0	0	1 (1.0)	0
Hyponatremia	0	0	0	0	0	0	0	0	0	0	0	0	1 (4.5)	1 (4.5)	1 (1.0)	1 (1.0)
Groin pain	0	0	0	0	0	0	0	0	0	0	0	0	1 (4.5)	0	1 (1.0)	0
Muscular weakness	0	0	0	0	0	0	0	0	0	0	0	0	1 (4.5)	0	1 (1.0)	0
Tumor pain	1 (6.7)	0	0	0	0	0	0	0	0	0	0	0	0	0	1 (1.0)	0
Dysgeusia	0	0	0	0	0	0	0	0	1 (25.0)	0	0	0	0	0	1 (1.0)	0
Memory impairment	0	0	0	0	0	0	0	0	1 (25.0)	0	0	0	0	0	1 (1.0)	0
Depression	0	0	0	0	1 (5.6)	0	0	0	0	0	0	0	0	0	1 (1.0)	0
Nephritis	0	0	0	0	1 (5.6)	0	0	0	0	0	0	0	0	0	1 (1.0)	0
Pleurisy	0	0	0	0	1 (5.6)	1 (5.6)	0	0	0	0	0	0	0	0	1 (1.0)	1 (1.0)
Pneumonitis	0	0	1 (6.7)	0	0	0	0	0	0	0	0	0	0	0	1 (1.0)	0
Bullous dermatitis	0	0	0	0	0	0	0	0	0	0	0	0	1 (4.5)	1 (4.5)	1 (1.0)	1 (1.0)
Papular rash	0	0	0	0	1 (5.6)	0	0	0	0	0	0	0	0	0	1 (1.0)	0
Urticaria	0	0	0	0	0	0	0	0	0	0	1 (10.0)	0	0	0	1 (1.0)	0
Embolism	0	0	0	0	1 (5.6)	1 (5.6)	0	0	0	0	0	0	0	0	1 (1.0)	1 (1.0)

Hot flush	0	0	0	0	0	0	0	0	0	0	0	0	1 (4.5)	0	1 (1.0)	0

[a]Adverse events by MedDRA preferred term in decreasing order of frequency (total column).
Abbreviations: Gr, grade; MedDRA, Medical Dictionary for Regulatory Activities; Q2W, every 2 weeks; Q4W every 4 weeks; TEAE, treatment-emergent adverse event.

Supplemental Table 9. Incidence and management of skin-related TEAEs in patients requiring concomitant medicines.

Pt /Dose cohort	Grade (Serious Y/N)	TEAE start and end: days from start of treatment	Dose change (Y/N)	Corticosteroid use: days since start of treatment	Outcome
**Pruritus**					
3.0 mg/kg Q2W	1 (N)	4, 57	N	N	Resolved
3.0 mg/kg Q2W	1 (N)	175, 204	N	N	Resolved
5.0 mg/kg Q2W	1 (N)	15, 24	N	Day 15, NA	Resolved
5.0 mg/kg Q2W	1 (N)	2, 7	N	Day 2, NA	Resolved
5.0 mg/kg Q2W	1 (N)	10, 29	N	Day 2, NA	Resolved
5.0 mg/kg Q2W	1 (N)	170, 174	NA	N	Resolved
400 mg Q4W	3 (N)	5, 7	N	Day 5, 8	Resolved
400 mg Q4W	2 (N)	8, 14	N	Day 8, 20	Resolved
400 mg Q4W	1 (N)	15, 22	N	Day 9, 25	Resolved
300 mg Q2W	1 (N)	8, NA	N	Day 67, NA	Ongoing
300 mg Q2W	1 (N)	10, 28	N	N	Resolved
5.0 mg/kg Q2W	1 (N)	15, NA	N	N	Ongoing
**Generalized pruritus**					
0.03 mg/kg Q2W	1 (N)	8, 17	N	N	Resolved
3.0 mg/kg Q2W	1 (N)	213, NA	N	Day 213, NA	Ongoing
5.0 mg/kg Q2W	1 (N)	25, NA	N	Day 15, NA	Ongoing
400 mg Q4W	1 (N)	7, 32	N	Day 10, 32	Resolved
300 mg Q2W	1 (N)	9, 19	N	Day 15, NA	Resolved
**Generalized rash**					
0.03 mg/kg Q2W	1 (N)	8, 26	N	N	Resolved
0.03 mg/kg Q2W	1 (N)	40, 50	N	N	Resolved
0.03 mg/kg Q2W	1 (N)	77, 84	NA	N	Resolved
3 mg/kg Q2W	2 (N)	128, 156	N	Day 128, NA	Resolved
3.0 mg/kg Q2W	1 (N)	213, NA	N	Day 213, NA	Ongoing
5.0 mg/kg Q2W	1 (N)	25, NA	N	Day 15, NA	Ongoing
10 mg/kg Q2W	2 (N)	99, 124	N	N	Resolved
400 mg Q4W	1 (N)	7, 32	N	Day 10, 32	Resolved

300 mg Q2W	1 (N)	9, 19	N	Day 9, NA	Resolved
**Rash maculo-papular**					
3.0 mg/kg Q2W	1 (N)	13,29	N	15, 29	Resolved
400 mg Q2W	1 (N)	107, ongoing	N	N	Ongoing

TRAE, treatment-related adverse event.

Supplemental Table 10. Grade ≥3 TRAEs and exposure.

Dose	Event	Grade	Serious Y/N	Event start date (days since start of treatment)	Treatment cycle
3 mg/kg Q2W	Cardiac tamponade	4	Y	10	1
3 mg/kg Q2W	Diarrhea	3	Y	10	1
3 mg/kg Q2W	Increased ALT	3	Y	10	1
3 mg/kg Q2W	Increased AST	3	Y	10	1
3 mg/kg Q2W	Cough	3	Y	10	1
3 mg/kg Q2W	Dyspnea	3	Y	10	1
3 mg/kg Q2W	Constrictive pericarditis	3	Y	11	1
3 mg/kg Q2W	Dyspnea	3	Y	53	During follow-up after cycle 1
3 mg/kg Q2W	Anemia	3	N	43	3
5 mg/kg Q2W	Pleurisy	3	N	20	2
5 mg/kg Q2W	Dyspnea	3	Y	39	2
5 mg/kg Q2W	Embolism	3	Y	50	During follow-up after cycle 2
20 mg/kg Q2W	Fatigue	3	N	91	5
400 mg Q4W	Pruritus	3	N	5	1
300 mg Q2W	Bullous dermatitis	3	N	40	3
300 mg Q2W	Fatigue	3	N	31	3
300 mg Q2W	Hyponatremia	3	N	16	2
300 mg Q2W	Fatigue	3	N	47	4
300 mg Q2W	Anemia	3	Y	36	3

TRAE, treatment-related adverse event.

Supplemental Table 11. Fatal TEAEs.

Dose	Cause of death	Time of fatal TEAE (days since start of treatment)	On treatment vs during follow-up	Date treatment discontinued due to progressive disease
0.03 mg/kg	Septic shock	21 days	On treatment	
0.03 mg/kg	Malignant neoplasm progression	56	Follow-up	51
0.1 mg/kg	Sepsis	113	Follow-up	71
0.1 mg/kg	Malignant neoplasm progression	16	Follow-up	15
10.0 mg/kg	Cardiac arrest	84	Follow-up	57
300 mg	Dyspnea	49	On treatment	
300 mg	Malignant neoplasm progression	64	Follow-up	49
300 mg	Malignant neoplasm progression	53	Follow-up	15[a]
300 mg	Malignant neoplasm progression	35	Follow-up	22
3.0 mg/kg	Malignant neoplasm progression	22	Follow-up	11
3.0 mg/kg	Cardiorespiratory arrest	29	On-treatment	
5.0 mg/kg	Malignant neoplasm progression	59	Follow-up	33
1.0 mg/kg	Malignant neoplasm progression	63	Follow-up	32
10.0 mg/kg	Brain edema/Respiratory failure	18	On treatment	
10.0 mg/kg	thrombotic stroke	75	Follow-up	57
5.0 mg/kg	Cardiogenic shock	45	Follow-up	3[b]
400 mg	Malignant neoplasm progression	65	Follow-up	57

[a] Reason for discontinuation not recorded; [b] Treatment discontinued due to serious TEAE of acute myocardial infarction. TEAE, treatment-emergent adverse event.

Supplemental Table 12. TEAEs leading to treatment discontinuation.

Dose cohort	Event	Treatment-related (Y/N)	Duration from treatment start to discontinuation (days)
3.0 mg/kg Q2W	Cardio-respiratory arrest	N	29
5.0 mg/kg Q2W	Hypersensitivity reaction	Y	57
5.0 mg/kg Q2W	Myocardial infarction	N	10
10.0 mg/kg Q2W	Large intestinal obstruction	N	177
10.0 mg/kg Q2W	Brain edema and respiratory failure	N	32

TEAE, treatment-emergent adverse event.

Supplemental Table 13. TEAEs leading to treatment interruption.

Dose		Events (n)		
	**Patients with events**	**Treatment interruptions**	**Treatment restarted**	**Adverse events relapsed**
0.1 mg/kg Q2W	1	1	1	0
0.3 mg/kg Q2W	1	2	2	1
3.0 mg/kg Q2W	3	5	2	0
5.0 mg/kg Q2W	4	4	4	0
10.0 mg/kg Q2W	2	4	2	0
20.0 mg/kg Q2W	2	6	1	0
300 mg Q2W	7	17	15	0

One patient in the 3.0 mg/kg Q2W group had three TEAEs requiring dose interruptions; all recovered/resolved. One patient in the 10.0 mg/kg Q2W group had three TEAEs requiring dose interruption; one recovered/resolved. One patient in the 20.0 mg/kg Q2W group had four TEAEs requiring dose interruptions; all four recovered/resolved; one patient had two TEAEs requiring dose interruption, both recovered/resolved. In the 300 mg Q2W group, one patient had four TEAEs requiring dose interruptions; one recovered/resolved; one patient had five TEAEs requiring dose interruptions of which two recovered/resolved; one patient had four TEAEs requiring dose interruptions of which all four recovered/resolved.
TEAE, treatment-emergent adverse event.

Supplemental Table 14. Descriptive statistics of the pharmacokinetic parameters following the first dose (cycle 1) or at steady state (cycle 6).

Dose	Visit	*n*	AUC_{inf} or AUC_{tau} (mg·h/L)	Half-life (h)	CL (L/h)	Vz (L)	*n*	C_{max} (mg/L)	t_{max} (h)
0.03 mg/kg	Cycle 1	4	78.7 (28.5), 75.1	80.6 (20.3), 78.6	0.0366 (0.0192), 0.0327	3.96 (1.63), 3.71	4	0.781 (0.135), 0.772	0.583 (0.533, 0.583)
	Cycle 6	0	-	-	-	-	-	-	-
0.1 mg/kg	Cycle 1	4	368 (126), 350	119 (32.0), 116	0.0189 (0.00445), 0.0186	3.12 (0.438), 3.09	4	2.70 (0.779), 2.61	0.608 (0.533, 0.917)
	Cycle 6	0	-	-	-	-	-	-	-
0.3 mg/kg	Cycle 1	3	1270 (238), 1260	180 (39.9), 177	0.0136 (0.00226), 0.0134	3.45 (0.236), 3.44	4	6.45 (1.33), 6.35	0.642 (0.583, 4.25)
	Cycle 6	1	1400	206	0.0105	3.14	1	7.82	0.583
1 mg/kg	Cycle 1	3	4450 (1110), 4360	171 (49.3), 167	0.0229 (0.00478), 0.0226	5.45 (0.567), 5.43	3	21.6 (0.321), 21.6	0.500 (0.500, 0.833)
	Cycle 6	1	7050	372	0.0150	8.04	1	36.5	4.00
3 mg/kg	Cycle 1	15	12,700 (4630), 12,000	165 (59.3), 155	0.0197 (0.00614), 0.0186	4.28 (1.09), 4.15	15	66.0 (13.7), 64.8	0.717 (0.550, 4.57)
	Cycle 6	5	21,400 (5880), 20,800	291 (78.1), 283	0.0127 (0.00565), 0.0117	5.03 (1.68), 4.78	7	109 (21.2), 107	0.583 (0.533, 4.08)
5 mg/kg	Cycle 1	16	24,900 (8260), 23,600	174 (44.5), 168	0.0175 (0.00460), 0.0170	4.27 (1.13), 4.14	17	128 (28.3), 125	0.633 (0.500, 4.70)
	Cycle 6	5	33,600 (16,700), 29,500	268 (120), 247	0.0168 (0.00702), 0.0158	5.74 (1.23), 5.62	6	182 (60.8), 173	0.683 (0.500, 4.92)
10 mg/kg	Cycle 1	14	55,900 (18,100), 53,400	179 (47.3), 172	0.0163 (0.00494), 0.0157	4.01 (0.949), 3.90	15	288 (70.8), 280	0.583 (0.500, 4.50)
	Cycle 6	4	76,900 (9630), 76,400	223 (7.31), 223	0.0138 (0.00137), 0.0137	4.43 (0.444), 4.42	5	436 (90.2), 428	0.667 (0.550, 4.00)
20 mg/kg	Cycle 1	3	103,000 (31,400), 99,500	191 (52.8), 186	0.0168 (0.00649), 0.0160	4.30 (0.447), 4.29	3	428 (70.5), 424	1.42 (0.650, 4.12)
	Cycle 6	0	-	-	-	-	-	-	-

300 mg	Cycle 1	21	21,000<br>(7290), 20,000	213<br>(156), 190	0.0156<br>(0.00451), 0.0150	4.28<br>(1.31), 4.10	22	102<br>(25.5), 99.4	0.667<br>(0.533, 4.67)
	Cycle 6	7	27,300<br>(11,000), 25,600	248<br>(100), 229[a]	0.0125<br>(0.00374), 0.0120	4.39<br>(0.882), 4.31[a]	10	140<br>(20.8), 139	0.675<br>(0.500, 4.72)
400 mg	Cycle 1	9	27,000<br>(14,000), 24,200	210<br>(95.5), 191	0.0181<br>(0.00750), 0.0165	4.65<br>(0.977), 4.56	10	127<br>(22.5), 126	0.558<br>(0.500, 4.50)
	Cycle 6	3	41,900<br>(19,300), 39,100	202, 263[b]	0.0154, 0.0110[b]	4.51, 4.16[b]	3	168<br>(28.5), 166	0.583<br>(0.583, 4.00)
Dose Independent PK Parameters	Cycle 6	25	NC	259<br>(84.2), 246‡	0.0137<br>(0.00457), 0.0130	4.91<br>(1.34), 4.74‡	26	NC	0.667<br>(0.5, 4.92)

Abbreviations: AUC_{inf}, area under the single-dose plasma or serum concentration-time curve extrapolated to time of infinity; AUC_{tau}, area under the single-dose plasma or serum concentration-time curve over the dosing interval; CL, total systemic clearance; C_{max}, maximum observed plasma concentration; NC, not calculated; PK, pharmacokinetic; t_{max}, time to maximum concentration; Vz, volume of distribution during the elimination phase.

Note: Summary values are presented as mean (STD), geometric mean except t_{max}, which is presented as median (min, max). All doses were Q2W except 400 mg, which was Q4W. At cycle 6 patients missing terminal timepoints were excluded from calculations of AUC_{tau}, CL, Vz, and half-life but were included in C_{max} and t_{max} calculations. However, the same patients were used for the summarization of t_{max} across doses as for the other PK parameters. This table includes only ADA negative individuals at the respective visit.

[a] $n = 6$; [b] $n = 2$; [c] $n = 24$.

Supplemental Table 15. Incidence of antidrug antibodies to INCAGN01876.

Dose	Number of patients with confirmed ADA/number of patients in dose group (%)
0.03 mg/kg Q2W	4/4 (100)
0.1 mg/kg Q2W	3/4 (75)
0.3 mg/kg Q2W	3/4 (75)
1 mg/kg Q2W	1/3 (33)
3 mg/kg Q2W	0/15 (0)
5 mg/kg Q2W	2/18 (11)
10 mg/kg Q2W	0/16 (0)
20 mg/kg Q2W	1/4 (25)
300 mg Q2W	1/22 (4.5)
400 mg Q4W	0/10 (0)

Abbreviations: ADA, antidrug antibodies; Q2W, every 2 weeks; Q4W, every 4 weeks.

Supplemental Table 16. Best overall responses based on RECIST v1.1 and mRECIST.

	Treatment group							
	**0.030 to 1 mg/kg Q2W (*n* = 15)**	**3 mg/kg Q2W (*n* = 15)**	**5 mg/kg Q2W (*n* = 18)**	**10 mg/kg Q2W (*n* = 16)**	**20 mg/kg Q2W (*n* = 4)**	**400 mg Q4W (*n* = 10)**	**300 mg Q2W (*n* = 22)**	**Total (*N* = 100)**
BOR, n (%)								
CR	0	0	0	0	0	0	0	0
PR	1 (6.7)	0	0	0	0	0	1 (4.5)	2 (2)
SD	2 (13.3)	5 (33.3)	7 (38.9)	5 (31.3)	2 (50)	6 (60)	7 (31.8)	34 (34)
PD	7 (46.7)	7 (46.7)	8 (44.4)	10 (62.5)	2 (50)	4 (40)	11 (50)	49 (49)
NE	5 (33.3)	3 (20)	3 (16.7)	1 (6.3)	0	0	3 (13.6)	15 (15)
Median (95% CI) duration of disease control (CR+PR+SD), days	169 (91–NE)	58.5 (36–226)	58 (53–109)	62 (59–113)	61 (NE–NE)	113 (15–172)	213 (54–NE)	61 (59–113)

BOR, best overall response; CI, confidence interval; CR, complete response; mRECIST, modified Response Evaluation Criteria in Solid Tumors; NE, not evaluable; PD, progressive disease; SD, stable disease; PR, partial response; RECIST, Response Evaluation Criteria in Solid Tumors.

Supplemental Table 17. Representativeness of study participants.

Cancer type(s)/subtype(s)/stage(s)/condition	Advanced solid tumors, advanced adenocarcinoma of endometrium, melanoma, non-small lung cell cancer (NSCLC) and renal cell cancer (RCC).
Sex	Incidence across *all* cancers is slightly higher in men (212.5 per 100,000) vs women (186.2 per 100,000) (1) and this increase is seen at almost all shared anatomical sites, potentially due to sex-related biological mechanisms, as well as lifestyle factor (2). Examples of solid tumors with a higher incidence in men than women include lung, stomach, bladder, and colorectal cancer, whilst incidence of breast and thyroid cancer is higher in females than males (3)(55).
Age	Age is one risk factor associated with incidence of solid tumors, and risk increases with age. Median age of cancer diagnosis in the US is reported to be 66 years across all cancers with some variation by cancer type, for example, ranging from 62 to 71 years for breast, prostate, colorectal, and lung cancer (4).
Race/Ethnicity	Incidence of some solid tumors is known to vary with race and ethnicity, such as melanoma (higher in non-Hispanic White vs other races/ethnicities) (5), prostate cancer is higher in non-Hispanic Black men vs other races/ethnicities (5). Recent US data also show mortality from cancer is higher in non-Hispanic Black men and women vs other races/ethnicities (6). US data show non-Hispanic White patients remain greatly underrepresented in phase 1 cancer clinical trials, with representation in Phase 1 trials worse than in Phase 3 trials (7,8).
Geography	Incidence rates of solid tumors is known to vary by geographical region due to factors such as environmental risk factor exposure as well as barriers to effective prevention, early detection, and curative treatments (1).
Other considerations	Because one of the secondary endpoints was preliminary efficacy, we included tumor types that are known be immunogenic and have proven to respond to checkpoint inhibitors (adenocarcinoma of the endometrium, melanoma, NSCLC, and RCC). Other factors impacting on the representativeness of study participants with solid tumors include genetic susceptibility, exposure to environmental carcinogens (e.g., pollutants), healthcare utilization and access, cultural and socioeconomical factors (9).
Overall study representativeness	This phase 1/2 study enrolled a small population of patients (n=100) in the US. Phase 1 included patients with a range of advanced tumors; phase 2 included patients with adenocarcinoma of the endometrium, melanoma, NSCLC, and RCC. These tumor types are representative of the most common solid tumors, are associated with greatest mortality, and are common tumors that are known to respond to checkpoint inhibitors (1,3). The study enrolled slightly more women than men, and the median age of patients is slightly lower than the median age of cancer diagnosis. Our representation of non-White patients is greater than

	is typical for cancer clinical trials (15% vs <10%), but the study still over represents White patients based on their representation in the US population (7,8).

1. Bray F, Laversanne M, Sung H, Ferlay J, Siegel RL, Soerjomataram I, et al. Global cancer statistics 2022: GLOBOCAN estimates of incidence and mortality worldwide for 36 cancers in 185 countries. CA Cancer J Clin 2024;74(3):229-63 doi 10.3322/caac.21834.
2. Jackson SS, Marks MA, Katki HA, Cook MB, Hyun N, Freedman ND, et al. Sex disparities in the incidence of 21 cancer types: Quantification of the contribution of risk factors. Cancer 2022;128(19):3531-40 doi 10.1002/cncr.34390.
3. World Health Organization. Cancer today. 2024.
4. National Cancer Institute. 2021 17 December 2024. Cancer causes and prevention: age and cancer risk <https://www.cancer.gov/about-cancer/causes-prevention/risk/age>. Accessed 2024 17 December 2024.
5. National Cancer Institute. 2024 17 December 2024. Recent Trends in SEER age-adjusted incidence rates, 2000-2021: Melanoma of the skin. <https://seer.cancer.gov/statistics-network/explorer/application.html?site=53&data_type=1&graph_type=2&compareBy=race&chk_race_6=6&chk_race_5=5&chk_race_4=4&chk_race_9=9&chk_race_8=8&rate_type=2&sex=1&age_range=1&stage=101&advopt_precision=1&advopt_show_ci=on&hdn_view=0&advopt_show_apc=on&advopt_display=2#resultsRegion0>. Accessed 2024 17 December 2024.
6. National Cancer Institute. 17 December 2024. Cancer stat facts: cancer disparities. <https://seer.cancer.gov/statfacts/html/disparities.html>. Accessed 2024 17 December 2024.
7. Dunlop H, Fitzpatrick E, Kurti K, Deeb S, Gillespie EF, Dover L, et al. Participation of Patients From Racial and Ethnic Minority Groups in Phase 1 Early Cancer Drug Development Trials in the US, 2000-2018. JAMA Netw Open 2022;5(11):e2239884 doi 10.1001/jamanetworkopen.2022.39884.
8. Goel S, Negassa A, Ghalib MH, Chaudhary I, Desai K, Shah U, et al. Outcomes Among Racial and Ethnic Minority Patients With Advanced Cancers in Phase 1 Trials: A Meta-Analysis. JAMA Netw Open 2024;7(7):e2421485 doi 10.1001/jamanetworkopen.2024.21485.
9. National Cancer Institute. December 18. Cancer Disparities. <https://www.cancer.gov/about-cancer/understanding/disparities>. Accessed 2024 December 18.

Supplemental Figures

Supplemental Fig. 1. Study design.

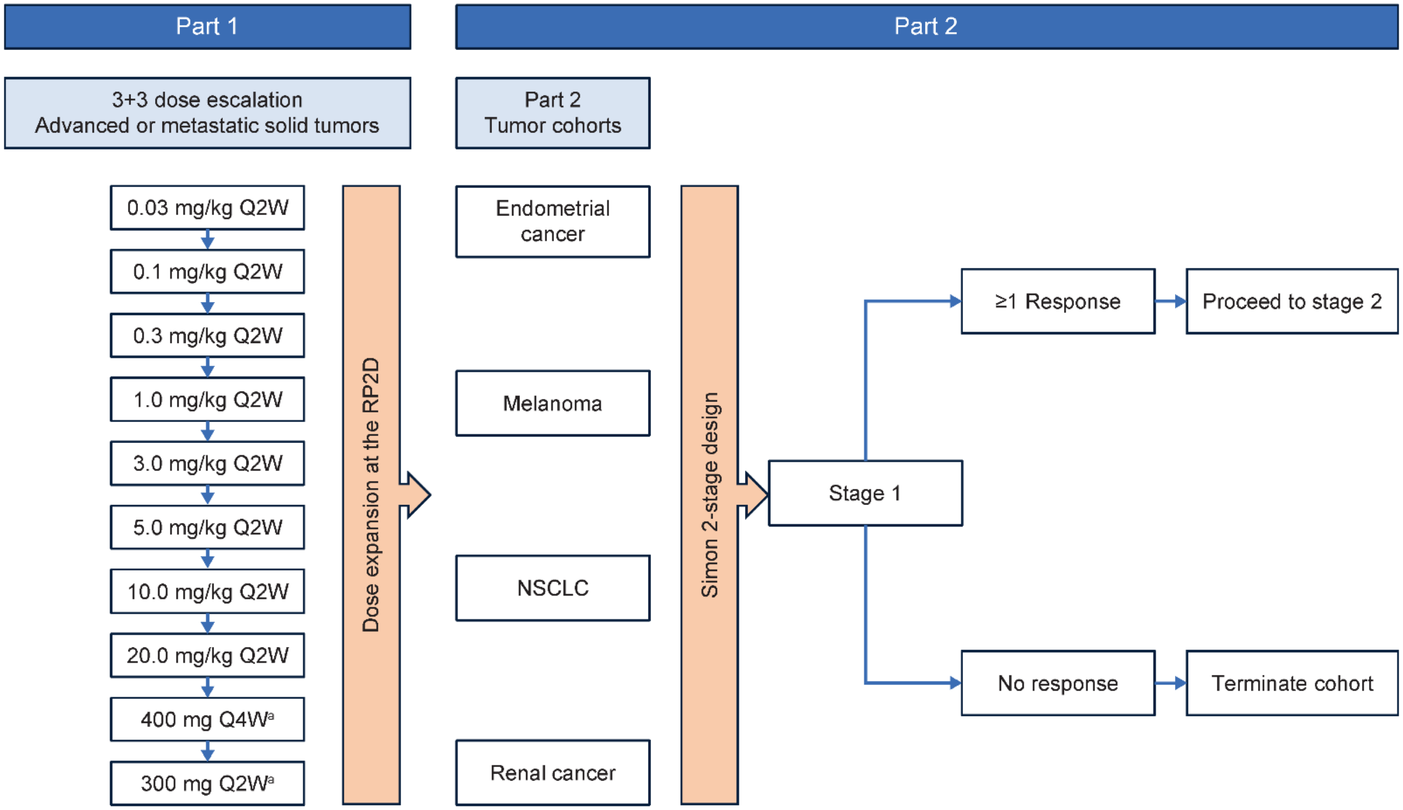

[a]Two post hoc flat doses of 400 mg every 4 weeks (Q4W), and 300 mg Q2W were assessed.
NSCLC, non-small cell lung cancer; Q2W, every 2 weeks; Q4W, every 4 weeks; RP2D, recommended part 2 dose.

Supplemental Fig. 2. INCAGN01876 individual predose trough concentrations versus time since first dose by dosing regimen.

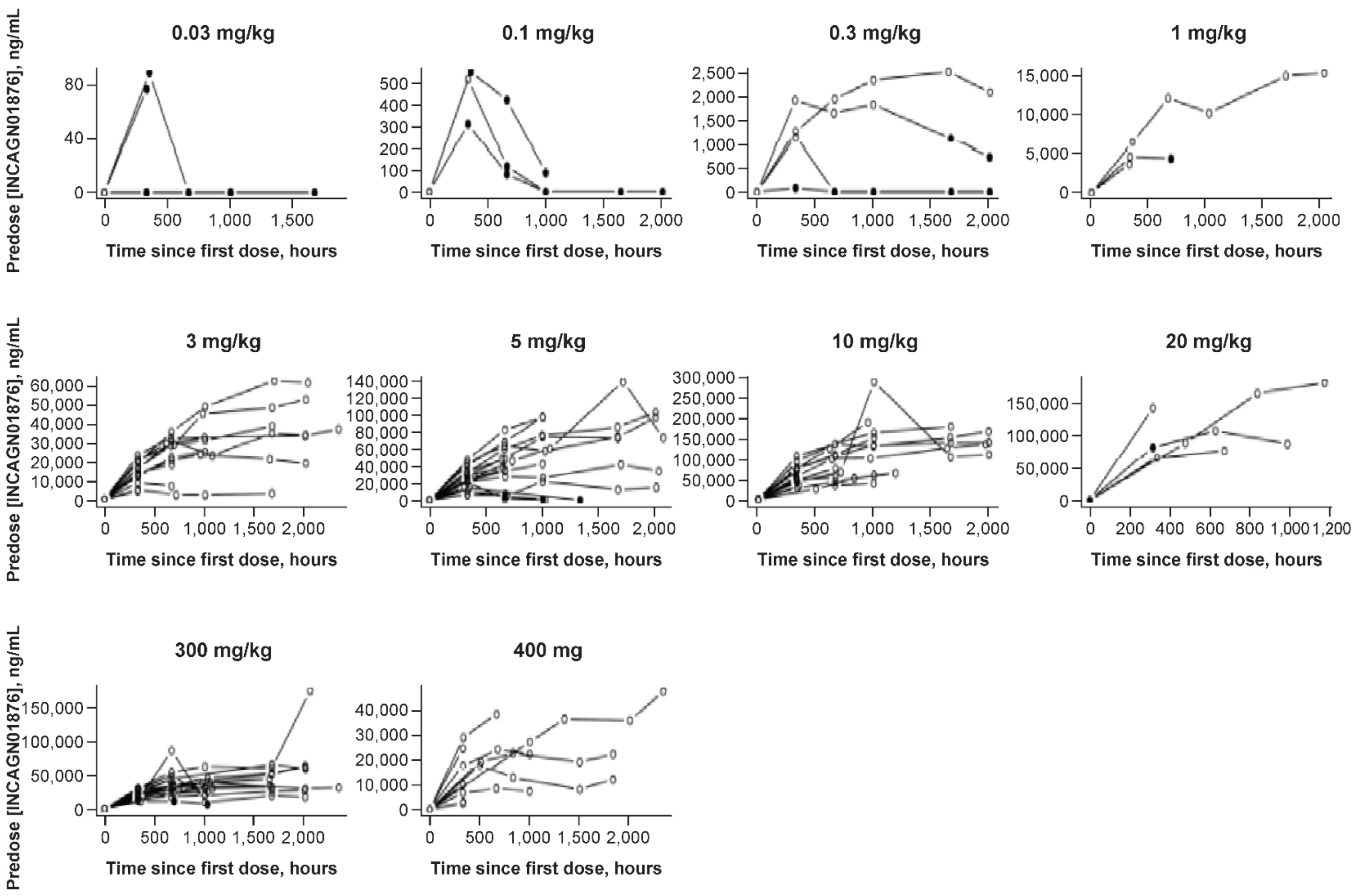

Closed points = ADA-positive; open points = ADA-negative. The sixth dose following Q2W dose administration occurred around 2016 hours. All doses were administered Q2W except 400 mg, which was Q4W.

Supplemental Fig. 3. Select INCAGN01876 individual concentration–time profiles at cycle 1 and cycle 6 depicting patients with ADA-positive and ADA-negative samples.

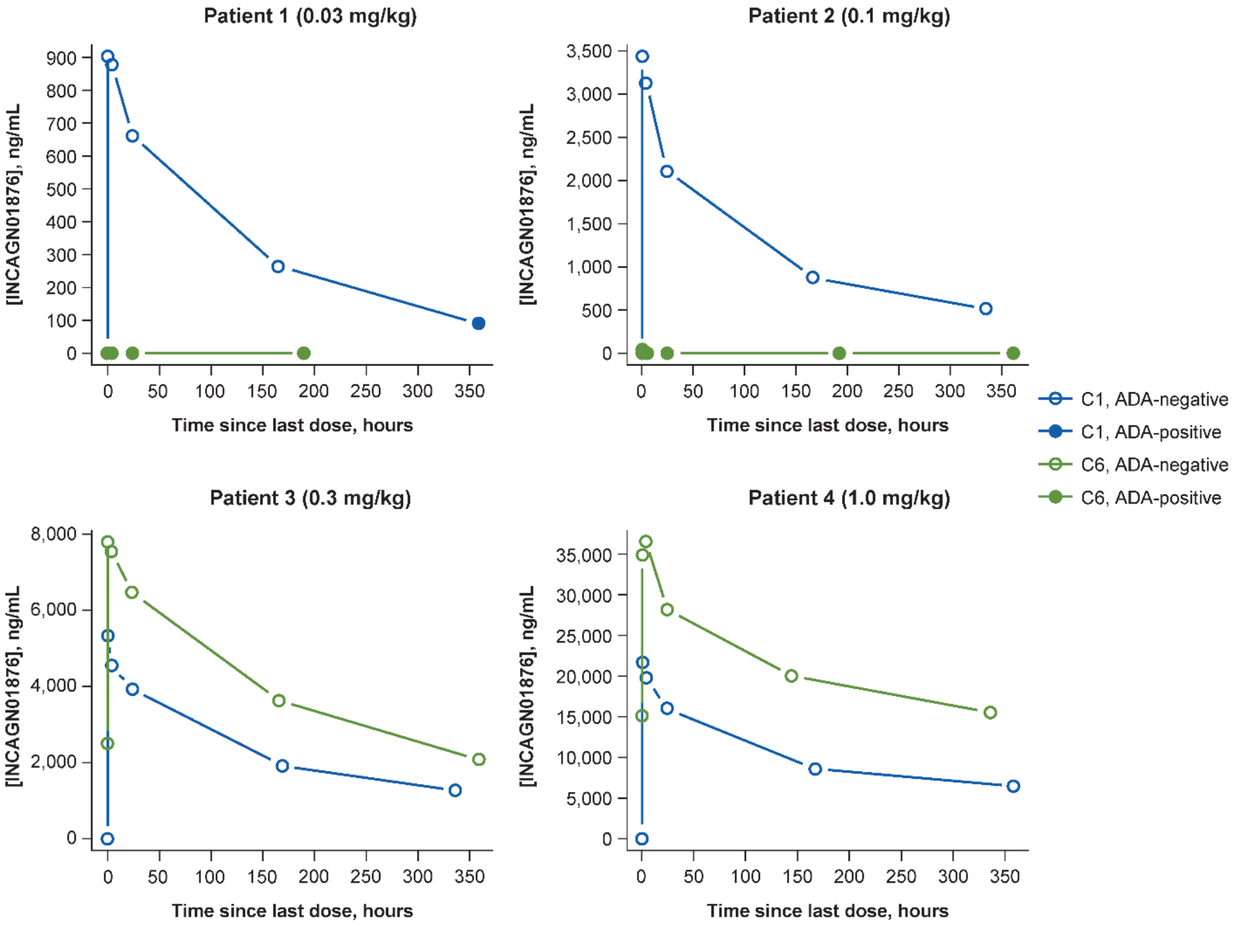

Supplemental Fig. 4. (A) Cytokine upregulation on INCAGN01876 treatment. (B) Relationship between INCAGN01876 dose and cytokine upregulation.

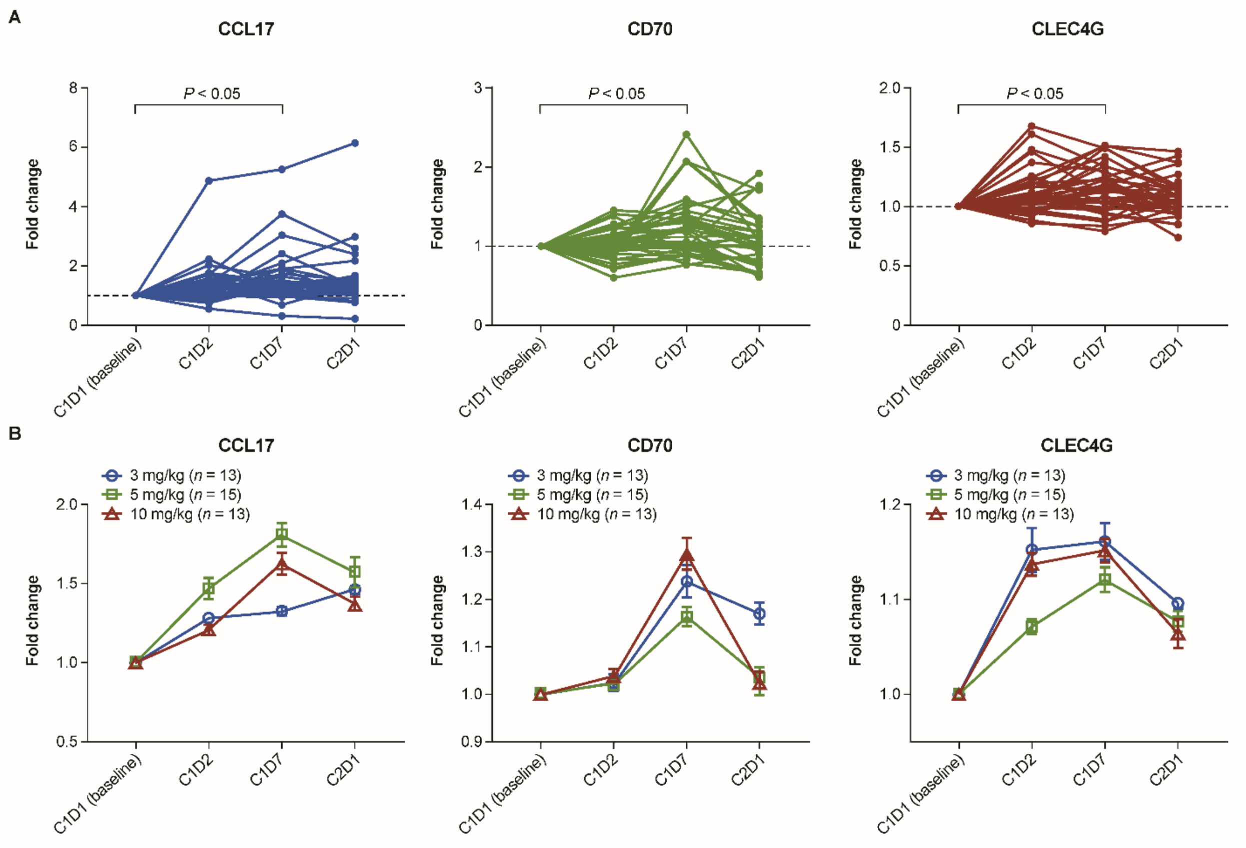

Relationships between INCAGN01876 dosing and dynamic change in CCL17, CD70 and CLEC4G upon treatment are depicted (with ±SE). Three dose curves are presented, with the number of patients in each cohort indicated.

Supplemental Fig. 5. Changes in frequency of (A) total and (B, D) GITR-expressing Tregs and (C, E) peripheral Ki67$^+$ CD8$^+$ T cells in the MSK cohort following INCAGN01876 treatment.

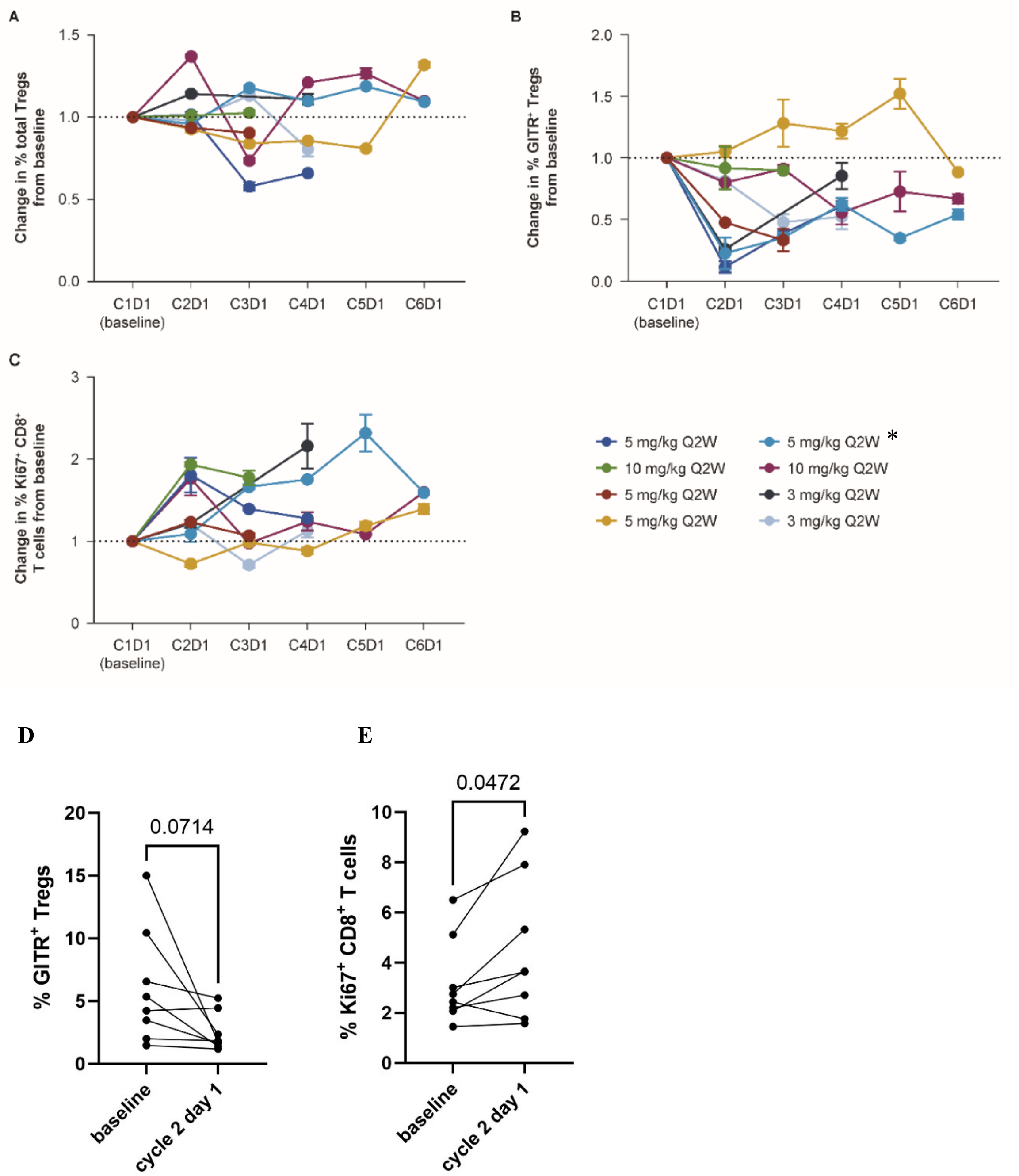

(A-C) Each colored line represents analyses for an individual patient. Average % change from baseline is depicted (± standard error of the mean). Each patient sample was run in duplicate, representing technical replicates. *Representative flow cytometry images for this patient are shown in Supplemental Fig 6 and 7. (D, E) Analysis of change from baseline to cycle 2 day 1 by two-tailed paired T-test, (D) percentage of GITR$^+$ Treg within the FoxP3$^+$ Treg population, (E) percentage of Ki67$^+$ CD8$^+$ T cells within the CD8$^+$ T cell population (E). Treg, regulatory T cell.

Supplemental Fig. 6. GITR^{+} FoxP3^{+} flow cytometry gating strategy data for GITR+FoxP3+ populations. Baseline examples are shown for the patient receiving INCAGN01876 5mg/kg Q2W indicated in Supplemental Figure 5.

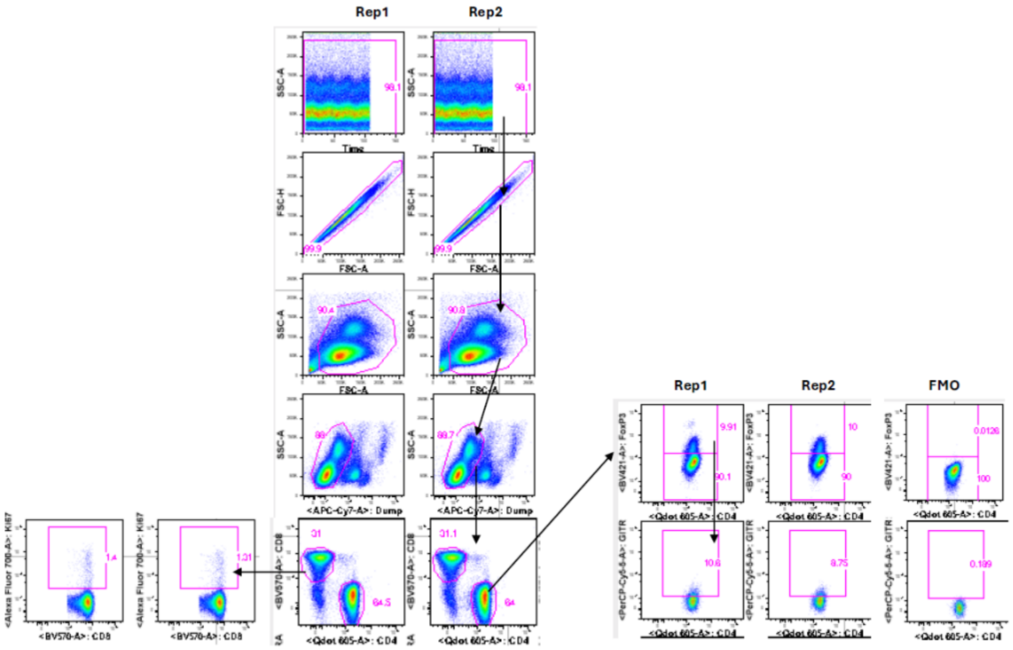

FMO, fluorescence minus one negative control; FSC-H, forward scatter-height; SSC-A, side scatter area; Rep, repeat.

Supplemental Fig. 7. Representative flow plots for GITR+ FoxP3+ cells showing reduction in GITR+ FoxP3+ cells over time from the patient receiving INCAGN01876 5 mg/kg Q2W indicated in Supplemental Figure 5.

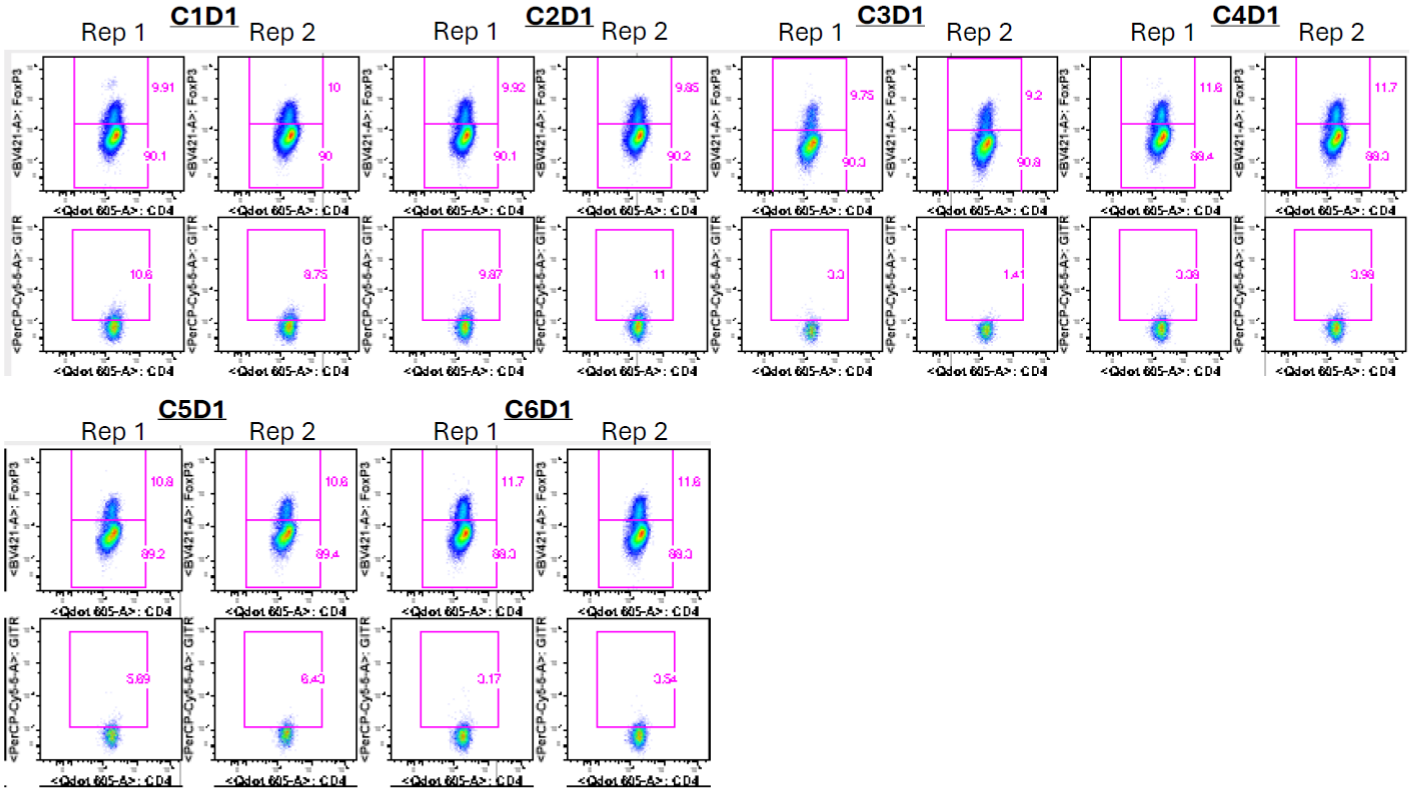

C, cycle; D, day; Rep, repeat.

Supplemental Fig. 8. Tumor infiltration of T cells in paired biopsies. Cell densities (cell numbers per mm^2) of various T-cell subsets in the tumor region are presented. Each colored line represents screening and post-treatment analyses for an individual patient. (A) CD3$^+$ T cell density, (B) CD4$^+$ T cell density, (C) Treg density, (D) CD8$^+$ T cell density, (E) proliferating T cell density, (F) activated T cell density.

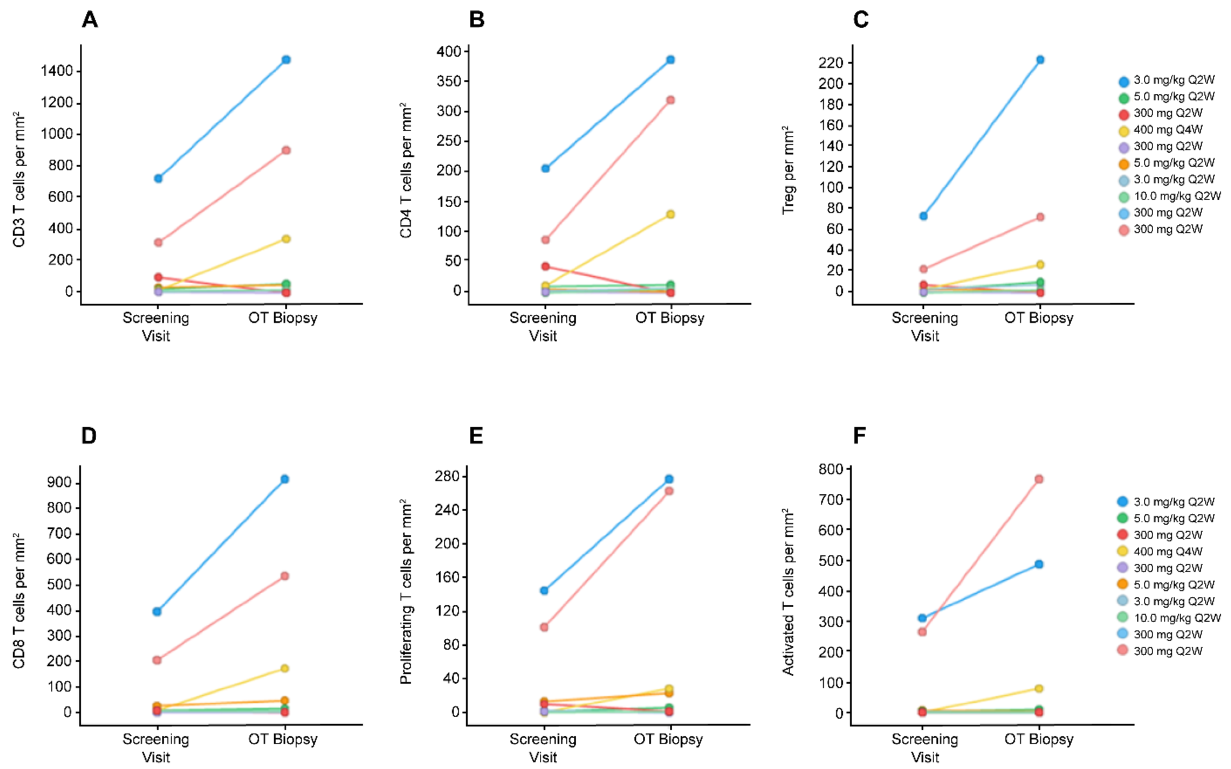

OT, on treatment; Treg, regulatory T cell.

Supplemental Fig. 9. Tumor infiltration of T cells in paired biopsies from MSK analysis. Cell densities (cell numbers per mm^2) of various T-cell subsets in the tumor region are presented. Each white bar represents screening and colored bars represent post-treatment analyses for each individual patient (1-7). (A) Treg density, (B) Ki67$^+$ Treg density, (C) GITR$^+$ Treg density, (D) CD8$^+$ T cell density, (E) Ki67$^+$ CD8$^+$ T cell density, (F) Teff density, (G) GITR$^+$ Teff density.

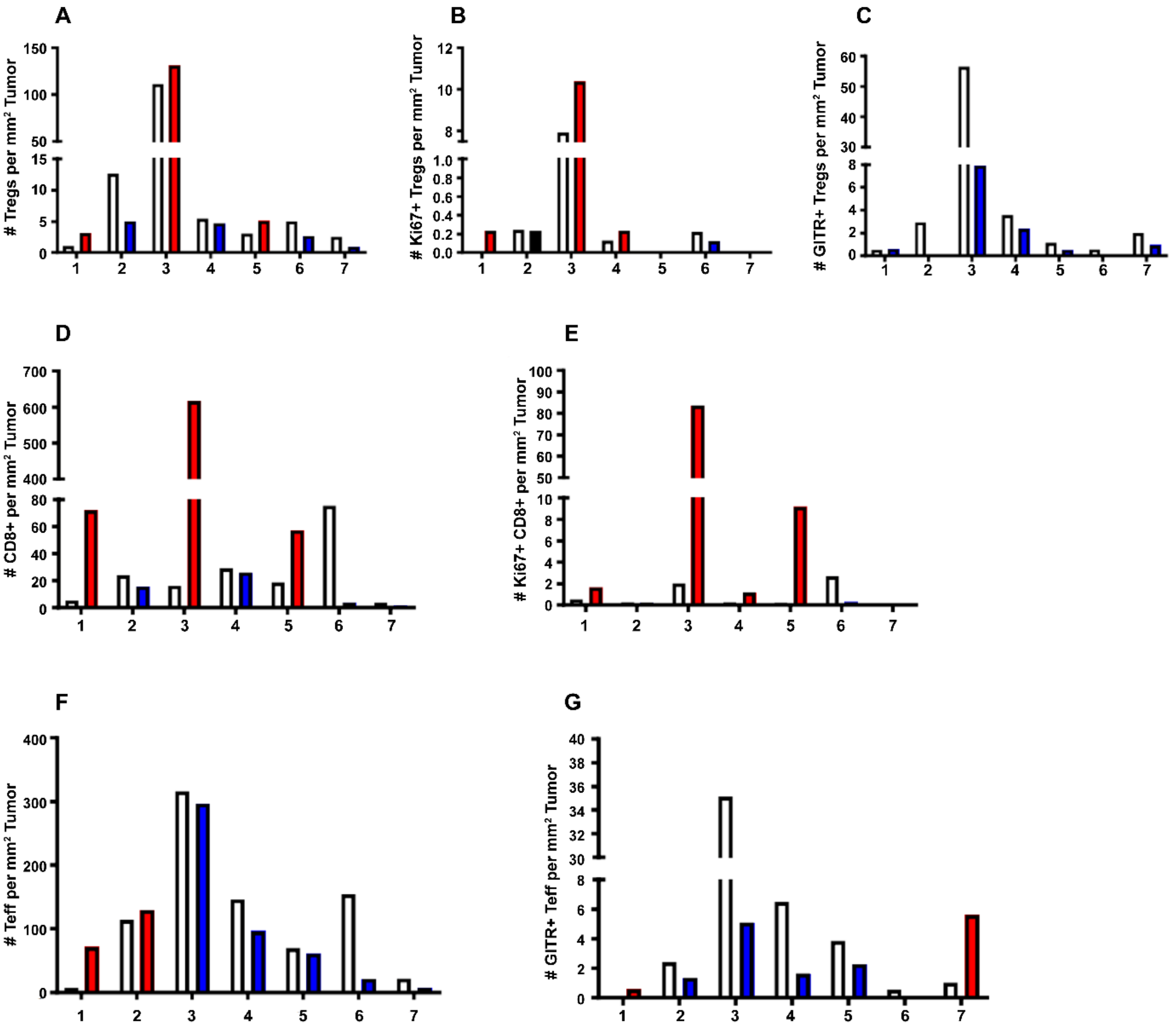

Teff, effector T cell; Treg, regulatory T cell.

Supplemental Fig. 10. Representative T cell infiltration biopsy immunofluorescence images from two patients, each showing two fields of view. (A) MultiOmyx staining results for a post-treatment biopsy performed at Incyte. (B) Representative images from pre- and post-treatment biopsies performed at MSK.

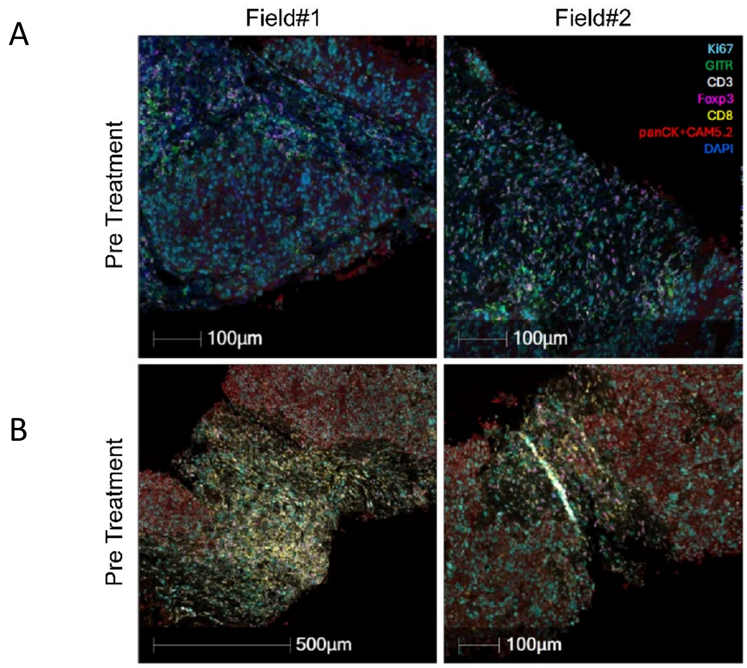

Supplement: Supplementary data1 — Supplementary materials [file ccr-24-4141_supplementary_data1_suppds1.pdf]
